# Youth Uptake of Digital Sexual and Reproductive Health Services Across Sociodemographic Groups (2018-2022): A Total Population Study from Stockholm, Sweden

**DOI:** 10.1016/j.mcpdig.2025.100251

**Published:** 2025-07-08

**Authors:** Lovisa Hellsten, Viktor H. Ahlqvist, Anna M. Nielsen, Gunnar Brandén, Anna Mia Ekström, Kyriaki Kosidou

**Affiliations:** a Department of Global Public Health, Karolinska Institutet, Stockholm, Sweden; b Institute of Environmental Medicine, Karolinska Institutet, Stockholm, Sweden; c Centre for Epidemiology and Community Medicine, Region Stockholm, Sweden; d Department of Biomedicine, Aarhus University, Denmark; e Department of Clinical Science and Education, Södersjukhuset, Karolinska Institutet, Stockholm, Sweden; f Department of Infectious diseases/Venhälsan, Södersjukhuset, Stockholm, Sweden

## Abstract

**Objective:**

To examine uptake of in-person and digital sexual and reproductive health (SRH) services among adolescents and young adults, quantify uptake across time, and explore whether the introduction of digital services affected the sociodemographic composition of users.

**Patients and Methods:**

This Swedish total population study included all Stockholm residents aged 12-22 years between January 1st 2018 and December 31st 2022. The primary outcome was in-person or digital visits (chat and video) of SRH services within a year, identified using regional health care registries. Sociodemographic predictors included sex, age, migrant background, parental education, and household income, analyzed with repeated-measures multivariable regressions.

**Results:**

Among the 454,405 individuals, 23.96% had at some point used SRH services (80.01% women) between 2018 and 2022. In-person visits remained the predominant mode of contact. Women had higher annual utilization rate of both in-person (women: 15.27%; 95% CI, 15.13-15.40; men: 1.75%; 95% CI, 1.72-1.78) and digital visits (women: 2.23%; 95% CI, 2.16-2.30; men: 0.12%; 95% CI, 0.11-0.13). Significantly lower uptake was also observed in the lowest income quintile (digital: adjusted odds ratio [aOR], 0.34; 95% CI, 0.31-0.36; in-person: aOR, 0.43; 95% CI, 0.42-0.45) compared with the highest quintile (reference group). Among digital visits, chat was more equitably used than video consultations across sociodemographic groups, including smaller differences between the highest and lowest income quintiles (chat: aOR, 0.59; 95% CI, 0.54-0.65; video: aOR, 0.25; 95% CI, 0.23-0.27). Only modest reductions in socioeconomic disparities were observed after the introduction of digital services.

**Conclusions:**

Sociodemographic disparities in utilization were not alleviated by the introduction of digital visits; in-person users were also the primary digital users. Chat could be more equitable than video, but further research is needed.

Telehealth is increasingly becoming an integral part of modern health care systems. Following disruptions to routine health care provision during the COVID-19 pandemic, digital modalities expanded rapidly.^1^^,^^2^ Youth represent a particularly promising demographic group for digital health care, given their high technological proficiency. A key priority in youth care is sexual and reproductive health (SRH) services, given the unique social, cognitive, and physical development needs characterizing adolescence and young adulthood.^3^ Digital service routes have gained momentum, and recent studies suggest that technology-based SRH services are perceived as confidential and private by youth, allowing for independent connections with health care providers while reducing barriers like cost and travel.^4^

Digital technologies have been suggested as a new determinant of health for youth, owing to their increasing importance.^1^ Meanwhile, digital solutions have been observed to be disproportionally accessed and adopted by more advantaged groups—this digital divide in combination with rapid changes to health care provision could exacerbate existing health inequalities.^5^^,^^6^ Despite the increasing use of digital technologies, the potential alleviation or amplification of sociodemographic inequalities in the young population remain understudied. Existing studies into digital SRH services for youth largely focus on perceptions and attitudes, use smaller patient populations, or address the application of digital solutions to SRH education and information.^4^^,^^7^, ^8^, ^9^, ^10^, ^11^, ^12^ Furthermore, comparisons of uptake across different digital modalities remain sparse, and it is unclear whether some might be more equitably accessed across sociodemographic groups than others.

In this study, we leverage Sweden’s unique health data context to examine the dynamics of digital SRH service use among youth. The Swedish setting is characterized by a universal health care system intended to minimize access barriers across age and sociodemographic groups,^13^^,^^14^ a long-standing commitment to SRH services specifically targeting youth,^15^ alongside a high level of digital maturity and ambition at societal level,^16^^,^^17^ and comprehensive national and regional registers that encompass the entire population.^18^ Through a total population analysis of adolescents and young adults (ages 12-22 years) residing in Stockholm, we aimed to identify uptake of in-person and digital visits across different sociodemographic groups, to quantify the uptake of digital SRH services within this demographic group across time, and to investigate whether the introduction of digital services has influenced the sociodemographic composition of users, potentially alleviating or exacerbating existing social gradients in SRH service access.

## Patients and Methods

This study is reported according to the Strengthening the Reporting of Observational Studies in Epidemiology guidelines (Supplemental Table 1, available online at https://www.mcpdigitalhealth.org/). The study was approved by the Swedish Regional Ethics Board (DNR: 2021–03075, 2023-02929-02, and 2024-01680-02).

### Study Population

Using pseudonymized Swedish personal identification numbers, assigned to each resident upon birth or immigration, we linked several regional health care and administrative registries to create an open cohort of all individuals aged 12-22 years residing in the Stockholm region between January 1, 2018, and December 31, 2022 (Supplemental Figure 1, available online at https://www.mcpdigitalhealth.org/). This resulted in 454,405 unique individuals eligible for inclusion in the analysis. No exclusions were made at the population level.

### Data

#### Stockholm Youth Clinic Services

Swedish youth clinics have offered confidential and free-of-charge services since the 1970s. In Stockholm, individuals aged 12 to 22 years have access to 31 youth clinics. Appointments include both SRH-related services (eg, prescriptions of contraceptives, sexually transmitted infection testing, emergency contraceptives, and free condoms) by medical staff and psychosocial support by social counselors.^7^

Digital SRH services were introduced at 1 selected youth clinic between October 2018 and March 2019 using video consultations. Following this pilot project, services were expanded to all youth clinics in Stockholm. Chat consultations—secure messaging—were introduced at selected clinics in April 2020 and made available in the entire region by November 2021. For the main analysis, we analyse digital uptake between January 1, 2020, and December 31, 2022. During the COVID-19 pandemic, drop-in visits on-site ceased at youth clinics and were reintroduced in the summer of 2022.

Video consultations are conducted in real-time (synchronously), whereas chat messaging enables users to respond at their convenience (asynchronously). All digital interactions are facilitated via the Stockholm region’s platform Always Open (Swedish: Alltid Öppet): a secure patient portal for digital health care contacts, accessed either via a smartphone application or website. Digital identification is required to access both video and chat consultations. A valid identity document is required to obtain digital identification, which for minors can only be issued with the consent of their legal guardian.

#### SRH Services

We retrieved data on SRH services—all consultations with medical staff (midwives, doctors, and nurses)—from youth clinics in the Stockholm region through the Regional Health Care Data Warehouse (VAL^19^) (Supplemental Table 2, available online at https://www.mcpdigitalhealth.org/). Information on chat consultations was sourced from the medical journal system TakeCare,^20^ covering all 28 publicly funded SRH services (chat data from 3 private clinics was not captured). We did not consider no-shows, atypical contacts (such as care without involvement of the patient), remote contacts (including telephone) aside from digital visits, due to incomplete data coverage of these modes of contact (Supplemental Figure 2, available online at https://www.mcpdigitalhealth.org/). This resulted in a final analytical sample of 492,773 contacts with medical staff from the total sample of 454,405 young individuals. We defined the primary outcomes as either an in-person visit or a digital visit, the latter encompassing both video and chat consultations as recorded in the previously mentioned data sources. Overall SRH service use encompasses both in-person and digital visits.

#### Sociodemographic Characteristics

The primary predictors of SRH service use included legal sex, age group (12-14, 15-19, and 20-22 years), migration status (Swedish-born parents, foreign-born parents, and foreign-born individuals), birth regions (Sweden, Europe, or rest of world), the highest level of parental education (primary education, secondary education, or postsecondary level), and birth year–specific quintiles of household disposable income recorded between 2018 and 2020. Parental linkages, sociodemographic information, and country of birth were sourced from registries maintained by Statistics Sweden.^21^, ^22^, ^23^ Detailed descriptions of each parameter can be found in Supplemental Table 3 (available online at https://www.mcpdigitalhealth.org/).

Data completeness was high across all individuals. However, 97,647 individuals (21.49% of the study population) had missing data on parental education and/or household income. Among overall SRH service users, the same data were missing for 9.27% of the individuals (Table). For all analyses, we used the missing-as-indicator approach and retained all individuals.^24^

**Table:** Sociodemographic Characteristics of the Study Population, Youth Aged 12-22 Y in Stockholm County 2018-2022, in Total and by Overall Sexual and Reproductive Health (SRH) Service Use at Youth Clinics

**Sociodemographic characteristics**	Total (N=454,405)	Users (n=108,895)	Nonusers (n=345,510)
Females	220,703 (48.57)	87,122 (80.01)	133,581 (38.66)
Males	233,702 (51.43)	21,773 (19.99)	211,929 (61.34)
Age (y), mean (SD)^a^	16.86 (3.18)	18.60 (2.28)	16.59 (3.21)
No. of contacts, mean (SD)^a^	0.31 (1.00)	2.29 (1.71)	NA
Migrant background			
Swedish-born parents	289,978 (63.81)	81,376 (74.73)	208,602 (60.38)
Foreign-born parents	67,463 (14.85)	12,940 (11.88)	54,523 (15.78)
Foreign-born	96,964 (21.34)	14,579 (13.39)	82,385 (23.84)
Birth region			
Sweden	357,441 (78.66)	94,316 (86.61)	263,125 (76.16)
Rest of Europe	30,617 (6.74)	4607 (4.23)	26,010 (7.53)
Rest of world	66,347 (14.60)	9972 (9.16)	56,375 (16.32)
Maternal education level			
Primary education	106,602 (23.46)	27,431 (25.19)	79,171 (22.91)
Secondary education	142,241 (31.30)	38,386 (35.25)	103,855 (30.06)
Postsecondary education	167,173 (36.79)	39,176 (35.98)	127,997 (37.05)
Missing	38,389 (8.45)	3902 (3.58)	34,487 (9.98)
Paternal education level			
Primary education	134,365 (29.57)	36,475 (33.50)	97,890 (28.33)
Secondary education	137,400 (30.24)	35,389 (32.50)	102,011 (29.52)
Postsecondary education	128,257 (28.23)	29,030 (26.66)	99,227 (28.72)
Missing	54,383 (11.97)	8001 (7.35)	46,382 (13.42)
Household income quintile			
Q1	84,623 (18.62)	17,172 (15.77)	67,451 (19.52)
Q2	77,111 (16.97)	20,699 (19.01)	56,412 (16.33)
Q3	75,495 (16.61)	22,054 (20.25)	53,441 (15.47)
Q4	76,030 (16.73)	22,721 (20.87)	53,309 (15.43)
Q5	76,689 (16.88)	23,265 (21.36)	53,424 (15.46)
Missing	64,457 (14.18)	2984 (2.74)	61,473 (17.79)

Values are n (%) unless specified.

a Interannual mean, 2018-2022.

### Statistical Analyses

First, descriptive characteristics of the overall study population, stratified by SRH service use over the study period, are presented. We then proceeded to model monthly trends in SRH service contacts (binary: contact or no contact within a given month per individual) using ordinary least squares regression with restricted cubic splines over time with knots placed at the 12th, 24th, 36th, 48th, and 60th month over the 5-year period. Data were aggregated by 10,000 inhabitants and analyzed separately by mode of contact.

To examine the sociodemographic characteristics of SRH service users, we used multivariable mixed-effects logistic regression models to estimate odds ratios and 95% CIs for each mode of contact. An annual repeated-outcome measure was used to assess the association between different levels of sociodemographic predictors and having an in-person visit, digital visit, and video or chat consultation. For enhanced public health and clinical interpretability, we also estimated the absolute standardized annual probability of a contact (hereafter annual utilization rate) from the aforementioned models. A random intercept at the individual level was incorporated to account for correlated outcomes within individuals. All analyses were adjusted for age (linear and squared term) and sex, except when these were the predictors. For all digital modalities, analyses were restricted to years 2020-2022.

We also examined shifts in the sociodemographic composition of SRH service users after a period of rapid health care digitalization. We calculated the change in overall SRH service use across sociodemographic groups, reporting the absolute percent and the marginal contrasts, before and after the COVID-19 pandemic. March 2020, marking the onset of the COVID-19 pandemic outbreak in Sweden, served as the cutoff for this comparison. For this analysis, we replace the annual outcome with a monthly outcome measure, to more closely align it with the COVID-19 outbreak.

As sensitivity analyses, all analyses at the population level were also stratified by sex (in-person and digital visits). We additionally repeated the primary analysis including only complete cases with full information on parental education and household income. All data processing, statistical analyses, and data visualizations were performed using Stata version 18.

### Ethical Statement

All research was performed in accordance with relevant guidelines/regulations. The study was approved by the Swedish Regional Ethics Board (DNR: 2021–03075, 2023-02929-02, and 2024-01680-02). Informed consent from study participants was not required because the Swedish Ethical Review Authority provide a waiver for the use of data from existing registers.^25^

## Results

### Descriptive Results

Among the 454,405 individuals aged 12 to 22 years residing in Stockholm County between 2018-2022, nearly one-fourth (23.96%) had used SRH services at youth clinics at some point (Table). Users were more often women, Swedish-born, and less likely to have parents from the lowest quintile of household disposable income. Women were, on average, younger than men (mean age, 18.49 vs 19.26 years) and tended to have more contacts per year (mean number of contacts per year, 2.39 [SD 1.76] vs 1.66 [SD 1.13]) (Supplemental Table 4, available online at https://www.mcpdigitalhealth.org/). Results presented further focus on the total population estimates, but sex stratified analyses are included in the Supplemental Table 4.

### Uptake of Digital SRH Services Across Time

**Figure 1:**
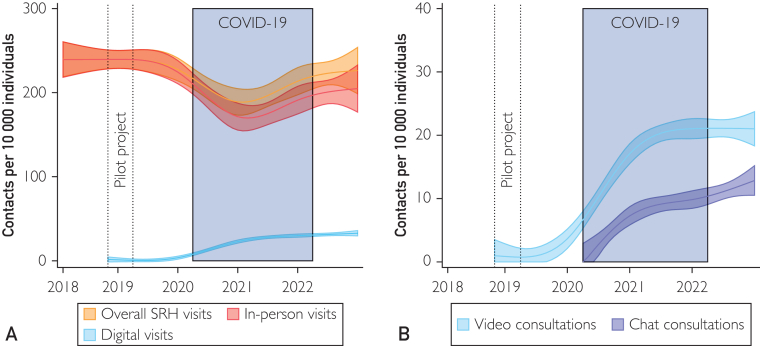
Estimated time trends in monthly sexual and reproductive health (SRH) service use among youth aged 12-22 years by mode of contact (A), and specifically among digital visits (B), 2018-2022 aggregated per 10,000 individuals. *Estimations using an ordinary least squares regression with restricted cubic splines with knots placed every year break (ie, 12-month distance).*

During the COVID-19 pandemic, SRH service use declined overall, but almost recovered to prepandemic levels in 2022 (Figure 1A; Supplemental Table 5, available online at https://www.mcpdigitalhealth.org/). In-person visits remained the most common mode of contact both before and after the COVID-19 pandemic. Of the digital visits, video and chat consultations increased in a nearly parallel manner, albeit with video consultations consistently showing higher uptake (Figure 1B). The trends were similar between men and women, albeit men potentially had a larger drop-in in-person visits during the pandemic (Supplemental Figure 3, available online at https://www.mcpdigitalhealth.org/).

### Digital SRH Service Use Across Sociodemographic Strata

**Figure 2:**
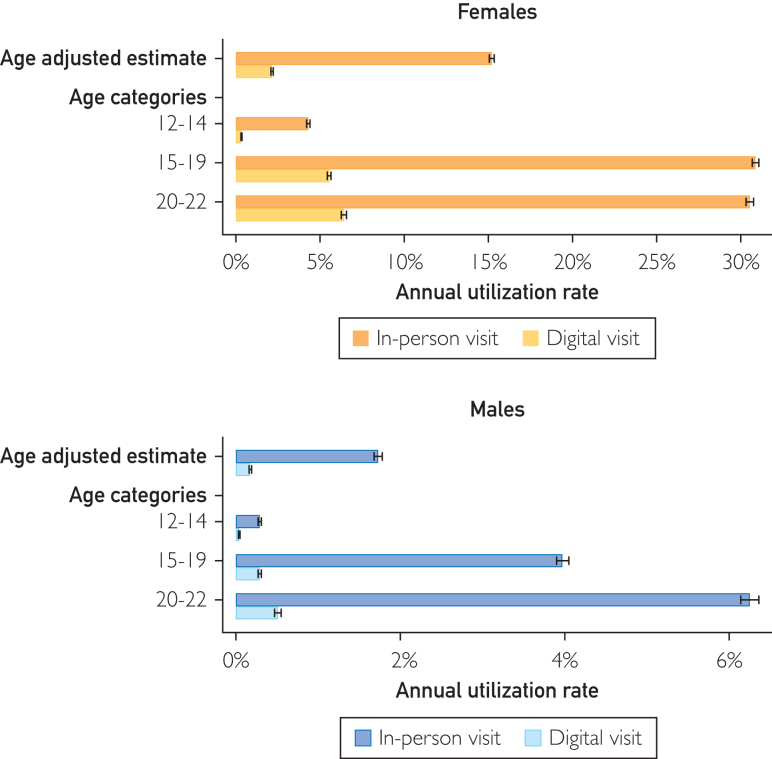
Sex stratified annual utilization rate of in-person and digital visits at the population level across the entire study period. *Sex was adjusted for age. Error bars represent 95% CIs.*

**Figure 3:**
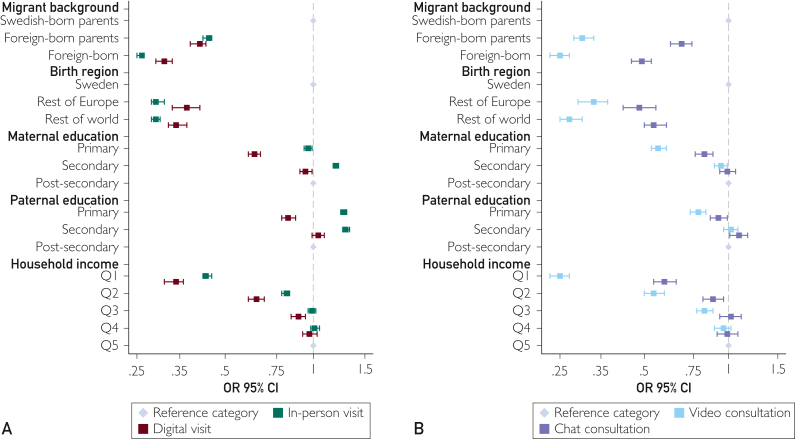
Adjusted odds ratios and 95% CIs of sexual and reproductive health (SRH) service contacts at the population level for in-person and digital visits (A) and chat and video (B) consultations, across the entire study period 2018-2022. *Estimates are adjusted for sex and age. Error bars represent 95% CI.*

After accounting for age differences, women had higher annual utilization rate of both in-person (15.27%; 95% CI, 15.13-15.40; vs men, 1.75%; 95% CI, 1.72-1.78) and digital visits (women, 2.23%; 95% CI, 2.16-2.30; vs men, 0.12%; 95% CI, 0.11-0.13) (Figure 2; Supplemental Figures 4 and 5, available online at https://www.mcpdigitalhealth.org/; Supplemental Tables 6-13, available online at https://www.mcpdigitalhealth.org/). Uptake of SRH services at population level were meanwhile lowest among men, the youngest age group, and lower income groups, as well as among youth themselves born outside of Sweden across all modes of contact (Figure 3A) Across parental education groups, in-person visits were least common in the upper secondary education group, whereas digital visits were the least common in the primary education group.

A social gradient was observed across both digital visits and in-person visits. Sex-adjusted and age-adjusted odds ratios (aORs) were highest in the reference groups with Swedish-born parents compared with those in the foreign-born parents (digital: aOR, 0.41; 95% CI, 0.38-0.43; in-person: aOR, 0.44; 95% CI, 0.42-0.45), when compared with the lowest quintile (digital: aOR, 0.34; 95% CI, 0.31-0.36; in-person: aOR, 0.43; 95% CI, 0.42-0.45). Across parental education groups, in-person uptake was highest in the mid-level (aOR, 1.19; 95% CI, 1.17-1.22; and 1.29, 95% CI 1.26-1.33, for maternal and paternal secondary education, respectively), whereas the odds of digital visits were lowest in the primary education group across both predictors (aOR, 0.63; 95% CI, 0.60-0.66; and aOR, 0.82; 95% CI, 0.78-0.87, for maternal and paternal education, respectively) (Supplemental Figure 6, available online at https://www.mcpdigitalhealth.org/; Supplemental Tables 14-17, available online at https://www.mcpdigitalhealth.org/).

Digital visits appeared more common among those with high socioeconomic status, but the sociodemographic pattern became more complex when separating digital visits into video and chat consultations (Figure 3B). Although the highest quintile of household income revealed the highest odds of both video and chat consultations, the difference between the highest and lowest quintile groups was smaller for chat consultations (aOR, 0.59; 95% CI, 0.54-0.65) than that for video consultations video (aOR, 0.25; 95% CI, 0.23-0.27) (Supplemental Tables 18-21, available online at https://www.mcpdigitalhealth.org/).

Sensitivity analyses restricted to complete cases produced similar results to the main analyses, with only marginal differences in point estimates (Supplemental Figure 7, available online at https://www.mcpdigitalhealth.org/; Supplemental Tables 22-27, available online at https://www.mcpdigitalhealth.org/).

### Changes in SRH Services Across Time Within Sociodemographic Strata

**Figure 4:**
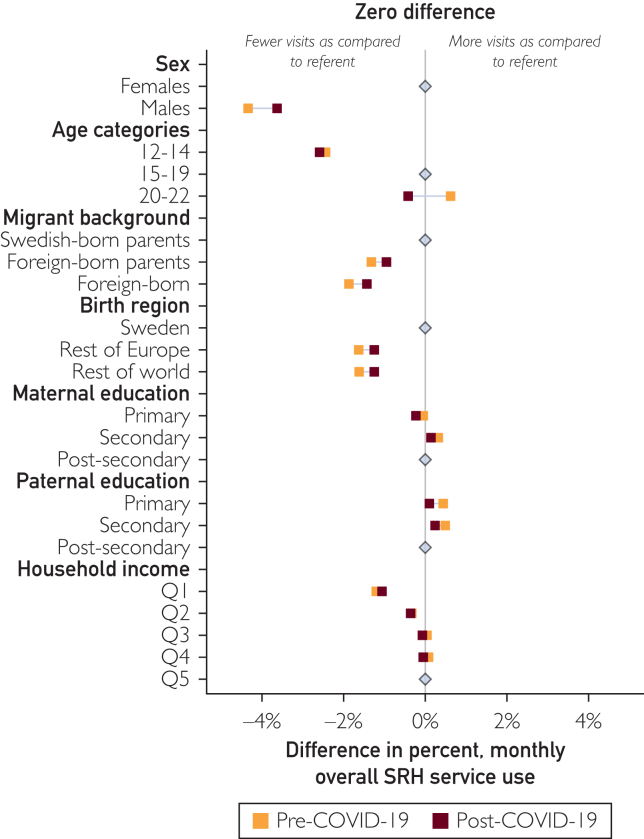
Adjusted differences in the percent of monthly overall sexual and reproductive health (SRH) service use at youth clinics across sociodemographic strata, before and after the COVID-19 pandemic, with estimates closer to zero signifying diminishing sociodemographic differences. *Estimates are adjusted for sex and age, except when analyzed as the strata. 95% CIs are plotted but, because of the large sample size, they are not visible beyond the point estimate but available in the supplement.*

Comparing overall SRH service use before and after the COVID-19 pandemic, we observed a marginal decrease in the relative differences in utilization across all strata, but the overall sociodemographic differences remained stable (Figure 4; Supplemental Table 28, available online at https://www.mcpdigitalhealth.org/). The most pronounced change was observed in the oldest age group (20-22 years), having higher utilization rates than the referent group (16-19 years) before the pandemic, but lower rates afterward. Men and the youngest age group consistently had lower overall SRH service use compared with women and the referent age group. Differences narrowed slightly in the postpandemic period, due to a significant decrease in utilization among women and the oldest age group (Supplemental Figure 8, available online at https://www.mcpdigitalhealth.org/; Supplemental Table 29, available online at https://www.mcpdigitalhealth.org/).

## Discussion

In this study of SRH service use in Stockholm, both in-person and digital SRH services were less commonly used by men, younger age groups, individuals from lower socioeconomic backgrounds, and those with a migrant background. Digital visits increased after the COVID-19 pandemic, but in-person visits remained the predominant mode of contact. The introduction of digital modes of contact did not alleviate disparities in overall SRH service use. Digital visits remain a small proportion of overall SRH services, but our findings suggest that asynchronous chat services could offer a more equitable uptake compared with synchronous video consultations.

### Previous Research in Digital SRH Services

To date, relatively few studies have examined sociodemographic characteristics and the uptake of digital SRH services among young people. Much of the existing quantitative research has focused on patient populations rather than population-based analyses. One exception is a population level study into SRH services among economically disadvantaged youth in Houston, Texas, which found telehealth visits (video and telephone) to be more common among women and higher-income patients.^26^ The same pattern was observed in a patient population of a California youth clinic.^8^ Although direct comparisons are challenging—given the context (Sweden vs the United States) and/or study design (patient-based vs total population)—the similarities in disparities to our findings are concerning. We also observed SRH service used to be lower among individuals with a migrant background, but the differences appeared smaller for chat contacts. Language barriers is a well-recognized challenge, and it may be that chat contacts are able to reduce communication difficulties compared with in-person visits.^2^^,^^7^^,^^8^^,^^26^ Previous research also suggests that migrant youth perceive youth clinics as less welcoming than Swedish-Scandinavian peers, citing concerns such as respect, equity, and parental support, and it could be that chats are able to mitigate some of these concerns.^27^

Few Swedish studies have examined digital SRH services, even outside the youth group. However, one cross-sectional study of 1016 women (aged 15-49 years) during the COVID-19 pandemic found that 14%-15% of the survey respondents had used telemedicine for contraceptive services and that contraceptive services were available via video consultations in 15 of 21 health care regions in Sweden.^28^ In a study comparing abortion regulation in Europe, Sweden also stood out as the only country that, before the COVID-19 pandemic, offered telemedicine for medical abortions.^29^ The overall aim of being a forerunner in digital welfare solutions, including health care, is echoed throughout the Swedish eHealth strategy.^17^ Digital solutions are also emphasized as a means of achieving the parallel goal of more patient-centered care nationally.^30^

### Persisting Sociodemographic Inequalities

Considering Sweden’s universal health care system and high digital readiness, the persistence of socioeconomic disparities observed is striking. Our findings suggest that even in a setting with (near) optimal conditions for digital SRH services, these were not equitably used by youth across sociodemographic groups.^2^^,^^31^^,^^32^ One explanation aligns with the so-called Nordic Paradox: despite universal welfare, socioeconomically advantaged groups tend to use and benefit more from health care services—including digital ones.^33^^,^^34^

In Sweden, low socioeconomic status among youth increasingly overlaps with a migrant background.^35^ This group may face additional challenges, such as differences in digital literacy and language barriers, which are known to hinder effective use of digital services.^36^^,^^37^ At youth clinics, midwives have reported that these language challenges are more pronounced in video consultations than in-person visits.^7^ Individuals born outside of Sweden report overall difficulties understanding and navigating the Swedish digital society, with compounding barriers including language, digital health literacy, and obtaining necessary electronic identification.^38^

Although digital access is widespread, 22% of foreign-born youth in deprived areas report being unable to afford the digital services they need—compared with 13% among Swedish-born youth.^38^ Despite high digital readiness at country and systemic levels, digital penetration might thus be slightly lower in some migrant youth populations. Finally, cultural norms, values, and attitudes may also reduce the perceived acceptability of SRH services overall, including for digital modes of contact, particularly where privacy cannot be ensured.^39^, ^40^, ^41^, ^42^, ^43^, ^44^

### Chat Contacts as More Equitable

The smaller differences observed for chat compared with those for video in our study highlight factors considered central to youth SRH services: privacy, confidentiality, and accessibility.^31^^,^^45^ Greater use of secure messaging services among youth has for instance been observed during months coinciding with increased SRH needs, potentially indicating lower thresholds to SRH counseling in sensitive matters.^46^ Although crowded housing and the risk of being overheard have been identified as barriers to telehealth including video,^7^^,^^47^ young people often perceive text-based SRH care as more anonymous, creating a less stigmatizing and more confidential space.^4^^,^^48^ This may be an important feature of chat consultations, where a more anonymous route to health care might also potentially serve as a more secure mode of contact for some groups than in-person visits or video consultations. When patient-provider contacts are increasingly digitalized, sensitive personal information like health care data—especially sexual health data—must, however, be ensured via a strong information security system, emphasizing the role of information technology security in digital health services.

Other key advantages of chat consultations include the ability to communicate outside of standard office hours, and less connectivity barriers compared to video consultations, which require stable internet access.^2^^,^^49^ However, the smaller disparities observed for chat compared with video consultations should be interpreted cautiously; there is a need for more data on this topic before any definitive conclusions can be made. Additionally, chat consultations remained highest among women, older ages, and higher socioeconomic groups, similar to user patterns in a young cohort from Washington state, assessing users of secure messaging.^50^

### Age, Sex, and Gender

Although sexual literacy, norms, and attitudes is established earlier in life, engagement in exploratory sexual activity and experiences of sexual relationship increase with age.^51^ Sexual and reproductive development increases during puberty, and the simultaneous cognitive development that occurs includes an increased interest in romantic and sexual relationships as well as exploring one’s sexual orientation.^31^ In Sweden, the mean age of sexual debut among youth in a recent survey-based population study was 16.7 (SD, 0.05) reflecting the higher uptake and presumed needs for SRH services observed in the older age groups of our study.^52^

Baseline SRH service needs are generally understood to vary by sex and age. However, the sex disparities observed may also reflect embedded gender norms influencing perceptions of acceptability and responsibility for SRH matters more broadly.^31^ Similar to SRH issues predominantly being considered a “woman’s issue,” Swedish youth clinics continue to be perceived of as girls’ clinics.^53^ Ensuring young men’s engagement in and access to SRH services nonetheless remain vital.^54^^,^^55^ In Sweden, men aged 16-29 years report lower rates of sexually transmitted infection testing and using more unreliable primary sources for SRH information than women.^52^ Men’s involvement in SRH has also been associated with improved contraceptive uptake and use among their female partners in heterosexual relationships.^56^ The limited engagement of men with SRH services observed could contribute to a gendered gap in SRH knowledge, reinforcing gendered responsibilities in heterosexual relationships.^57^ Although comprehensive sexuality education is an essential source of information for all youth, its role may potentially be of particular importance for young men's SRH understanding.

### Strengths and Limitations

This study has notable strengths, including its population-based sample and high-resolution analysis of a critical age groups for SRH service use including early adolescence. Furthermore, the ability to distinguish between video and chat consultations represents a key novelty because most previous studies of digital health care uptake have focused on the former or overall telehealth modalities.

This study also has several limitations. First, we were unable to assess whether adolescents sought SRH services outside youth clinics or examine whether content of care varied depending on modality. Second, the urban study setting is linked to higher youth clinic density and shorter travel distances. Digital uptake could be more pronounced in rural areas with greater travel distances. Third, digital health access is also influenced by health systems and digital infrastructure, which could further influence the degree of disparities globally.^1^^,^^2^ Fourth, our study captures the early phase of digital SRH service implementation, ending in 2022. As digital modalities become more integrated into routine care, the sociodemographic patterns observed may evolve, necessitating further longitudinal research. Fifth, Sweden’s comparatively light COVID-19 restrictions may have influenced SRH service use among youth relative to other countries.^58^^,^^59^ Despite restrictions on physical drop-in visits, it is unclear if stricter national measures would have increased digital service use or just reduced overall access to care. Future research should examine these dynamics in various policy contexts.

Finally, although our study provides early insights into the digitalization of youth clinics, there are several questions that will need further investigation as the digitization expands—including but not limited to its potential implications for health equity, resource allocation, and health care safety.^1^^,^^47^ Although the full impact of digitalizing youth clinics remains to be evaluated, our findings indicate persistent sociodemographic disparities in uptake. To address these, policymakers and public health practitioners may need to account for the digital determinants of health when planning and implementing digital health solutions.^60^^,^^61^ Importantly, in-person visits still constitute the majority of service use, highlighting the need to maintain accessible on-site care—particularly for groups less likely to engage with digital platforms. Nevertheless, because digital modes of contact have become politically and economically attractive, targeted efforts to increase uptake among disadvantaged groups may be warranted to more effectively promote such services.

## Conclusion

In this study of SRH service use in Stockholm, digital visits (chat and video consultations) increased after the COVID-19 pandemic but continue to represent a small proportion of overall SRH service use. Sociodemographic disparities in service utilization were not alleviated by the introduction of digital visits; women and higher socioeconomic groups, who represent the primary higher users of in-person services, were also the main digital users. Among digital visits, chat consultations could have a more equitable uptake across sociodemographic strata compared with video consultations. Further research is needed.

## Potential Competing Interests

The authors report no competing interests.

## Ethics Statement

All research was performed in accordance with relevant guidelines/regulations. The Swedish Ethical Review Authority provide a waiver for the use of data from existing registers, therefore informed consent from study participants was not required. The study was approved by the Swedish Regional Ethics Board (DNR: 2021–03075, 2023-02929-02, and 2024-01680-02).
