# Supplementary material for: Youth Uptake of Digital Sexual and Reproductive Health Services Across Sociodemographic Groups (2018-2022): A Total Population Study from Stockholm, Sweden
Source: Mayo Clin Proc Digit Health. 2025 Jul 8;3(3):100251. doi: 10.1016/j.mcpdig.2025.100251 (PMC12332933; doi:10.1016/j.mcpdig.2025.100251)
Supplement: Supplementary Tables [file mmc1.docx]

# Supplements

Supplemental Table 1. STROBE Statement—Checklist of items that should be included in reports of cohort studies

|  | Item No | Recommendation |
| --- | --- | --- |
| **Title and abstract** | 1 | Indicate the study’s design with a commonly used term in the title or the abstract  *Youth Uptake of Digital Sexual and Reproductive Health Services Across Sociodemographic Groups (2018-2022): A Total Population Study from Stockholm, Sweden* |
|  |  | (*b*) Provide in the abstract an informative and balanced summary of what was done and what was found  *Abstract.* |
| Introduction | | |
| Background/rationale | 2 | Explain the scientific background and rationale for the investigation being reported  *Introduction (p. 5).* |
| Objectives | 3 | State specific objectives, including any prespecified hypotheses  *Introduction (p. 5):*  *Through a total population analysis of adolescents and young adults (ages 12–22) residing in Stockholm, we aim to identify uptake of in-person and digital visits across different sociodemographic groups, to quantify the uptake of digital SRH services within this demographic across time, and to investigate whether the introduction of digital services has influenced the sociodemographic composition of users, potentially alleviating or exacerbating existing social gradients in SRH service access.* |
| Methods | | |
| Study design | 4 | Present key elements of study design early in the paper  *Methods (p.5-8).* |
| Setting | 5 | Describe the setting, locations, and relevant dates, including periods of recruitment, exposure, follow-up, and data collection.  *Methods (p.5-8): study population, data, and Sexual and Reproductive Health Contacts.* |
| Participants | 6 | (*a*) Give the eligibility criteria, and the sources and methods of selection of participants. Describe methods of follow-up.  *Supplemental Figure 1 (flow-chart) in supplements and study population (p. 6) in the methods section: Using pseudonymized Swedish personal identification numbers, assigned to each resident upon birth or immigration, we linked several regional healthcare and administrative registries to create an open cohort of all individuals aged 12–22 residing in the Stockholm region between January 1, 2018, and December 31, 2022. This resulted in 454,405 unique individuals eligible for inclusion in the analysis. No exclusions were made at the population level.* |
|  |  | (*b*) For matched studies, give matching criteria and number of exposed and unexposed |
| Variables | 7 | Clearly define all outcomes, exposures, predictors, potential confounders, and effect modifiers. Give diagnostic criteria, if applicable.  *Method, statistical analysis section.* |
| Data sources/ measurement | 8* | For each variable of interest, give sources of data and details of methods of assessment (measurement). Describe comparability of assessment methods if there is more than one group.  *See Supplemental Table 2 (p. 9-11 in supplement) for a detailed description of variables, their sources, and coding.* |
| Bias | 9 | Describe any efforts to address potential sources of bias.  Methods: Primary analyses repeated with complete cases – Supplemental tables 22-27 and Supplemental Figure 7. Analyses at the population level repeated stratified by sex, Figure 3, Supplemental Tables 10-13, and Supplemental Figure 6. |
| Study size | 10 | Explain how the study size was arrived at.  *Methods, study population, and Figure 1 in supplements (flow chart of birth cohorts and study population).* |
| Quantitative variables | 11 | Explain how quantitative variables were handled in the analyses. If applicable, describe which groupings were chosen and why.  *Detailed description of age categories in Supplemental Table 2. Grouping is derived from broader categories based on Sawyer et. al. 2012:*  *- early adolescence 10-14 years*  *- late adolescence 15-19 years*  *- early adulthood 20-24* |
| Statistical methods | 12 | (*a*) Describe all statistical methods, including those used to control for confounding  *Methods section, statistical analysis* |
|  |  | (*b*) Describe any methods used to examine subgroups and interactions  *Methods section, statistical analysis* |
|  |  | (*c*) Explain how missing data were addressed  *Methods section, statistical analysis* |
|  |  | (*d*) If applicable, explain how loss to follow-up was addressed |
|  |  | (*e*) Describe any sensitivity analyses  *Methods section, statistical analysis* |
| Results | | |
| Participants | 13* | (a) Report numbers of individuals at each stage of study—eg numbers potentially eligible, examined for eligibility, confirmed eligible, included in the study, completing follow-up, and analysed  *Supplemental Figure 1 - flowchart of individuals in the study population and annual populations.* |
|  |  | (b) Give reasons for non-participation at each stage  *No exclusions were made at population level.* |
|  |  | (c) Consider use of a flow diagram  Supplemental Figure 1. |
| Descriptive data | 14* | (a) Give characteristics of study participants (eg demographic, clinical, social) and information on exposures and potential confounders  *Table 1* |
|  |  | (b) Indicate number of participants with missing data for each variable of interest  *Table 1.* |
|  |  | (c) Summarise follow-up time (eg, average and total amount)  N/A |
| Outcome data | 15* | Report numbers of outcome events or summary measures over time  Supplemental Table 2 & 5. |
| Main results | 16 | (*a*) Give unadjusted estimates and, if applicable, confounder-adjusted estimates and their precision (eg, 95% confidence interval). Make clear which confounders were adjusted for and why they were included.  *See all unadjusted and adjusted estimates (sex, age, age^2^) and sex-stratified estimates in supplement.* |
|  |  | (*b*) Report category boundaries when continuous variables were categorized  *Detailed description of age categories in Supplemental Table 2. Grouping is derived from broader categories based on Sawyer et. al. 2012:*  *- early adolescence 10-14 years*  *- late adolescence 15-19 years*  *- early adulthood 20-24* |
|  |  | (*c*) If relevant, consider translating estimates of relative risk into absolute risk for a meaningful time period  *See Figure 2, Supplemental Figure 4-5, and Supplemental Table 6-13 for absolute risk (annual utilization rate), unadjusted and adjusted for sex, age and age^2^.* |
| Other analyses | 17 | Report other analyses done — e.g. analyses of subgroups and interactions, and sensitivity analyses  *Sex-stratified analyses and complete case analyses are presented in the supplement.* |
| Discussion | | |
| Key results | 18 | Summarise key results with reference to study objectives.  *First section in discussion.* |
| Limitations | 19 | Discuss limitations of the study, taking into account sources of potential bias or imprecision. Discuss both direction and magnitude of any potential bias  *Strengths and limitations in discussion.* |
| Interpretation | 20 | Give a cautious overall interpretation of results considering objectives, limitations, multiplicity of analyses, results from similar studies, and other relevant evidence.  *Discussion.* |
| Generalisability | 21 | Discuss the generalisability (external validity) of the study results.  *Strengths and limitations in discussion.* |
| Other information | | |
| Funding | 22 | Give the source of funding and the role of the funders for the present study and, if applicable, for the original study on which the present article is based.  *Funding statement.* |


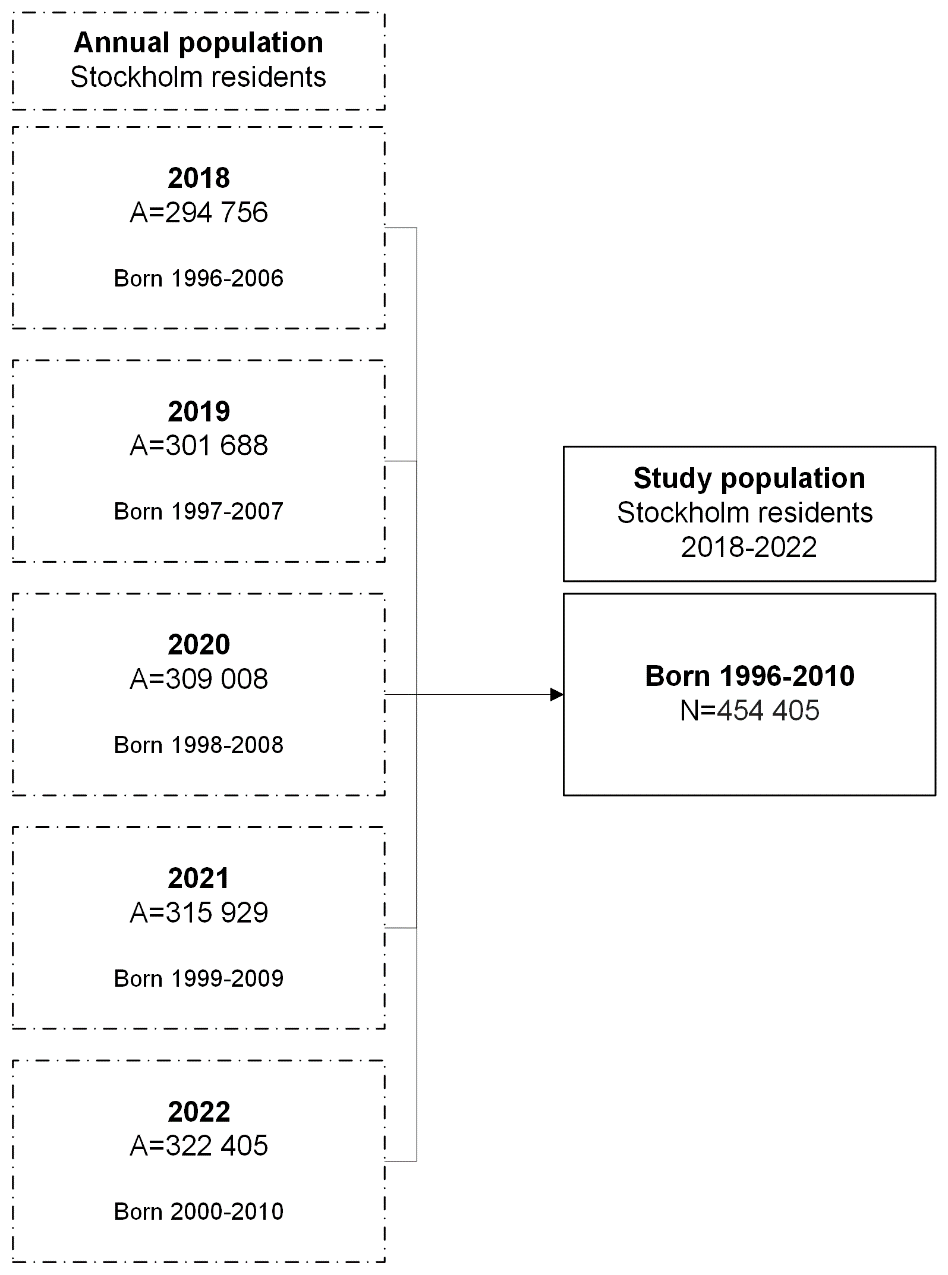


Supplemental Figure 1. Annual populations and study population, Stockholm residents 2018-2022.

Supplemental Table 2. Frequencies of Sexual and Reproductive Health (SRH) contacts at Youth Clinics in Region Stockholm 2018-2022 by mode of contact, in total and by sex.

| Mode of contact | Total | Females | Males |
| --- | --- | --- | --- |
| Mean number of annual contacts | 98 554.6 | *88 821* | *9733.6* |
| Overall SRH contacts | *492 773* | *444 105* | *48 668* |
|  |  |  |  |
| In-person | 474 149 | 426 423 | 47 726 |
| Digital | 31 271 | 29 577 | 1 694 |
| Video | 21 425 | 20 988 | 437 |
| Chat | 9 846 | 8 589 | 1 257 |


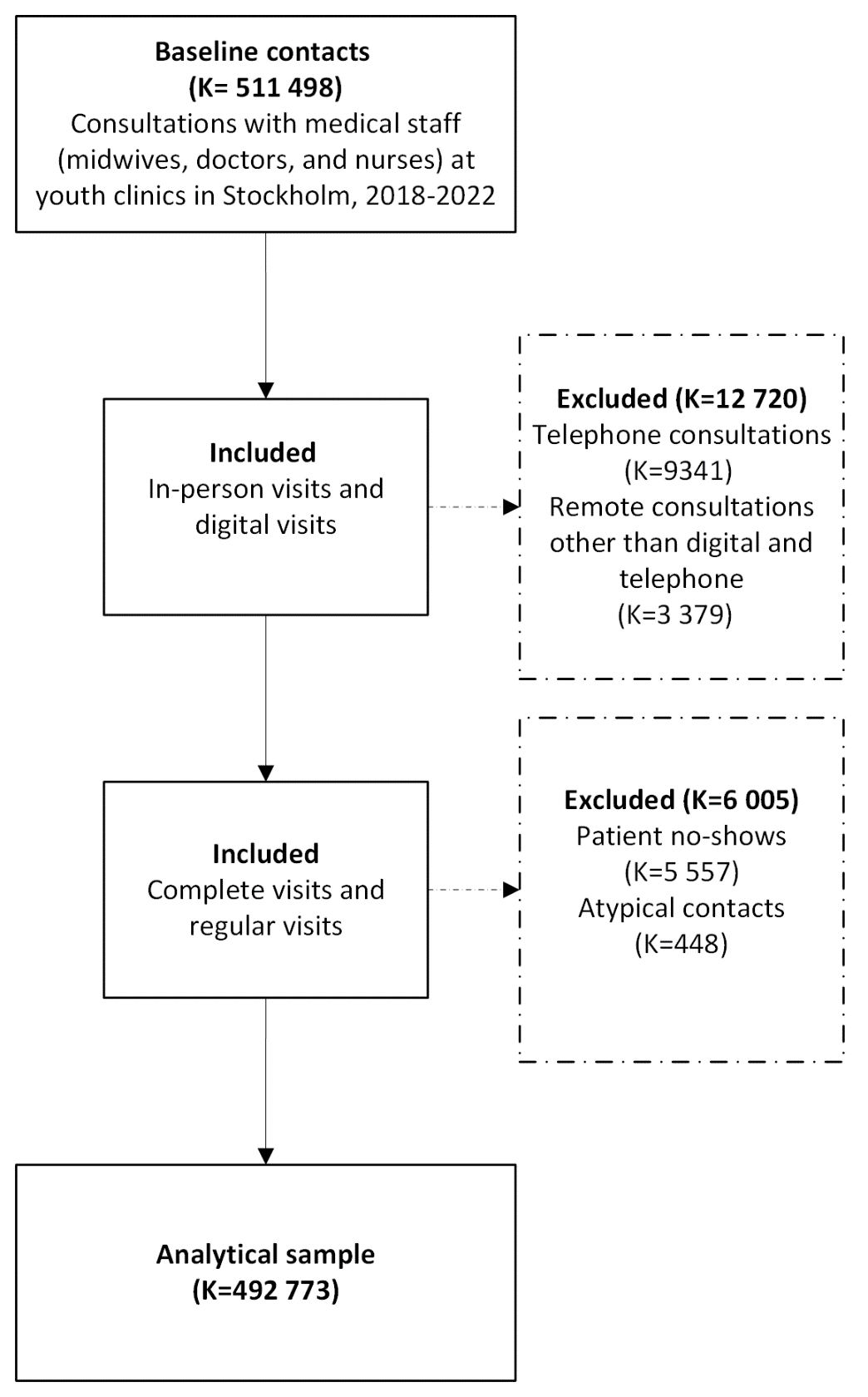


Supplemental Figure 2. Flow-chart of included contacts at youth clinics in Stockholm, 2018-2022.

Supplemental Table 3. Variable description and sources.

| **Variable** | | **Comment** | **Source** |
| --- | --- | --- | --- |
| ***ID*** | *A project ID is used to identify unique individuals in the dataset. These are derived from a pseudonymized version of the personal identification number (PIN) of all individuals registered in the Stockholm region*^1^ | *Statistics Sweden* | |
| **Sex**   1. Females 2. Males | | Individuals that have changed legal sex are not traceable, as their personal identification number is also changed in national registries. | Statistics Sweden^2-4^ |
| **Age categories**   1. 12-14 2. 15-19 3. 20-22 | | Age was categorized according to definitions presented in "Adolescence: A foundation for future health" (2012) by Sawyer et. al. in the panel *Definitions of adolescence and young adulthood (p. 1632):*   - *10–14 years (early adolescence)* - *15–19 years (late adolescence)* - *20–24 years (young adulthood)* | Statistics Sweden^2-4^ |
| **Migrant background**   1. Swedish-born parents 2. Foreign-born parents 3. Foreign-born | | Derived from classifications of migrant background used by Swedish authorities like [Statistics Sweden](https://www.statistikdatabasen.scb.se/pxweb/sv/ssd/START__BE__BE0101__BE0101Q/UtlSvBakgFin/table/tableViewLayout1/?loadedQueryId=113580&timeType=from&timeValue=2020).  Group (1) includes youth born in Sweden with either: two Swedish-born parents, one Swedish- and one foreign-born parent, or one identified parent identified born in Sweden.  Group (2) includes youth born in Sweden, with either: two foreign-born parents, or one identified parent born outside of Sweden.  Group (3) includes all youth born outside of Sweden. Aside from youth with own migration experience, this includes adoptees and youth born outside of Sweden with Swedish-born parents. | Statistics Sweden^2-4^ |
| **Birth region**   1. Born in Sweden 2. Born in rest of Europe 3. Born in rest of world | | Based on individual country of birth. | Statistics Sweden^2-4^ |
| **Maternal education**   1. Primary education 2. Upper secondary education 3. Post-secondary education | | Based classifications of educational degree available 2018-2020.   - *Primary education*    - Pre-primary education   - Primary and lower secondary education, (<9 years)   - Primary and lower secondary education (>=9 years)   - Upper secondary education (<2 years) - *Upper secondary education*    - Upper secondary education (>=2 years)   - University education (<2 years) - *Post-secondary education*    - Higher education, (>=2 years)   - Postgraduate/doctoral education | Statistics Sweden^2-4^ |
| **Paternal education** | | See information for maternal education above. | Statistics Sweden^2-4^ |
| **Household income** (quintiles, 1-5) | | Weighted income of parents adjusted for the birth year of offspring. The year with the maximum income level was used. Information available 2018-2020. | Statistics Sweden^2-4^ |
| ***Outcome variables*** | *Included visits:*   - All appointments with medical staff:   - Midwives   - medical doctors   - Nurses - Types of visits included:   - New visit, individual   - Outpatient visit, individual   - Video consultations   Chat consultations | Regional Health Care Data Warehouse (VAL)^5^  TakeCare (electronic health record system)^6^ | |

1. Ludvigsson JF, Otterblad-Olausson P, Pettersson BU, Ekbom A. The Swedish personal identity number: possibilities and pitfalls in healthcare and medical research. Eur J Epidemiol. 2009 Nov 6;24(11):659–67.
2. Ludvigsson JF, Almqvist C, Bonamy AKE, Ljung R, Michaëlsson K, Neovius M, et al. Registers of the Swedish total population and their use in medical research. Eur J Epidemiol. 2016 Feb 14;31(2):125–36.
3. Ekbom A. The Swedish Multi-generation Register. In 2011. p. 215–20.
4. Ludvigsson JF, Svedberg P, Olén O, Bruze G, Neovius M. The longitudinal integrated database for health insurance and labour market studies (LISA) and its use in medical research. Eur J Epidemiol. 2019 Apr 30;34(4):423–37.
5. Rajan G, Syding S, Ljunggren G, Wändell P, Wahlström L, Philips B, et al. Health care consumption and psychiatric diagnoses among adolescent girls 1 and 2 years after a first-time registered child sexual abuse experience: a cohort study in the Stockholm Region. Eur Child Adolesc Psychiatry. 2021 Nov 1;30(11):1803–11.
6. Cars T, Wettermark B, Malmström RE, Ekeving G, Vikström B, Bergman U, et al. Extraction of Electronic Health Record Data in a Hospital Setting: Comparison of Automatic and Semi‐Automatic Methods Using Anti‐Therapy as Model. Basic Clin Pharmacol Toxicol. 2013 Jun 7;112(6):392–400.

Supplemental Table 4. Sex-stratified sociodemographic characteristics of overall Sexual and Reproductive Health (SRH) service users, youth ages 12-22 in Stockholm County 2018-2022.

|  | Female users  (N=87 122) | Males users  (N=21,773) | p-value (χ² Test)* |
| --- | --- | --- | --- |
| Mean age, (SD)^1^ | 18.49 (2.29) | 19.26 (2.09) |  |
| Mean number of contacts, (SD)^1^ | 2.39 (1.76) | 1.66 (1.13) |  |
| Migrant background |  |  |  |
| Swedish-born parents | 66 902 (76.79%) | 14 474 (66.48%) | <0.00 |
| Foreign-born parents | 9 597 (11.02%) | 3 343 (15.35%) |  |
| Foreign-born | 10 623 (12.19%) | 3 956 (18.17%) |  |
| Birth region |  |  |  |
| Sweden | 76 499 (87.81%) | 17 817 (81.83%) | <0.00 |
| Rest of Europe | 3 705 (4.25%) | 902 (4.14%) |  |
| Rest of world | 6 918 (7.94%) | 3 054 (14.03%) |  |
| Maternal education level (2018-2020) |  |  |  |
| Primary education | 21 412 (24.58%) | 6 019 (27.64%) | <0.00 |
| Secondary education | 31 252 (35.87%) | 7 134 (32.77%) |  |
| Post-secondary education | 31 968 (36.69%) | 7 208 (33.11%) |  |
| Missing | 2 490 (2.86%) | 1 412 (6.49%) |  |
| Paternal education level (2018-2020) |  |  |  |
| Primary education | 28 956 (33.24%) | 7 519 (34.53%) | <0.00 |
| Secondary education | 28 836 (33.10%) | 6 553 (30.10%) |  |
| Post-secondary education | 23 563 (27.05%) | 5 467 (25.11%) |  |
| Missing | 5 767 (6.62%) | 2 234 (10.26%) |  |
| Household income (2018-2020) |  |  |  |
| Q1 | 13 153 (15.10%) | 4 019 (18.46%) | <0.00 |
| Q2 | 16 596 (19.05%) | 4 103 (18.84%) |  |
| Q3 | 17 952 (20.61%) | 4 102 (18.84%) |  |
| Q4 | 18 651 (21.41%) | 4 070 (18.69%) |  |
| Q5 | 18 826 (21.61%) | 4 439 (20.39%) |  |
| Missing | 1 944 (2.23%) | 1 040 (4.78%) |  |
|  |  |  |  |
| *Individuals missing information on parental education and/or household income.* | 7 389 (8.48%) | 2 701 (12.41%) |  |

^1^ Interannual mean, 2018-2022.* Note: p-values reflect differences between **users** and **non-users** within each sex category over the study period.

Supplemental Table 5. Sexual and Reproductive Health (SRH) service users in the annual population by year and mode of contact.

| **Year** | **2018** | **2019** | **2020** | **2021** | **2022** | **Total** |
| --- | --- | --- | --- | --- | --- | --- |
| **Overall SRH-service users* (%)** | 44,146 (14.98) | 44,376 (14.71) | 40,778 (13.20) | 40,788 (12.91) | 44,730 (13.87) | 214,818 (13.92) |
| **In-person users (%)** | 44,151 (14.98) | 44,300 (14.68) | 40,451 (13.09) | 38,550 (12.20) | 42,235 (13.10) | 209,687 (13.58) |
| **Digital users**  **(%)** | 39(0.01) | 539 (0.18) | 3,813 (1.23) | 8,178 (2.59) | 9,290 (2.88) | 21,859 (1.42) |
| **Annual population count (%)** | 294,756 (100) | 301,688 (100) | 309,008 (100) | 315,929 (100) | 322,405 (100) | 1,543,786 (100) |

*Definition of users: individuals with minimum 1 registered visit in a designated year.


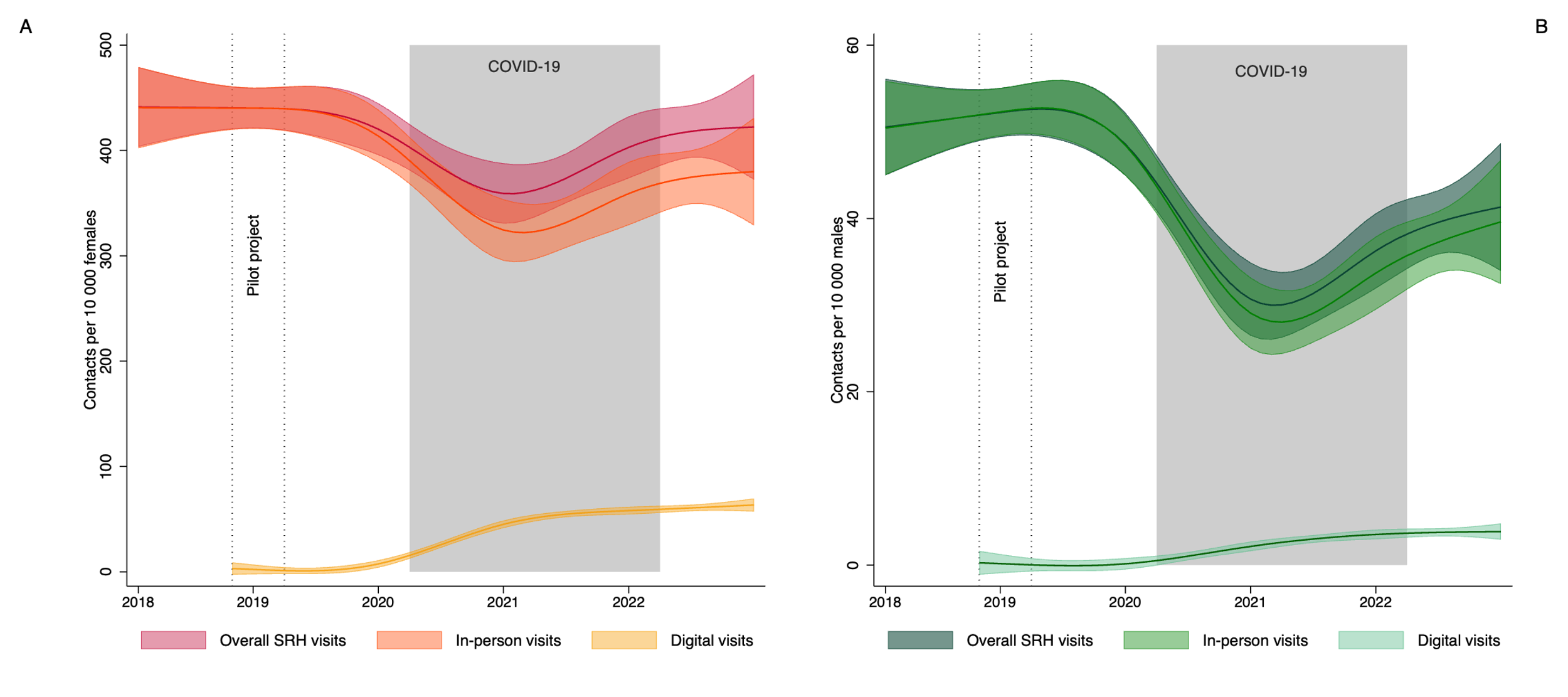


Supplemental Figure 3. Estimated time trends in monthly Sexual and Reproductive Health (SRH) service use by mode of contact among youth 12-22 in females (A) and males (B), 2018-2022 aggregated per 10,000 individuals. Estimated using a ordinary least squares regression with restricted cubic splines with knots placed every year break (i.e., 12-months distance).


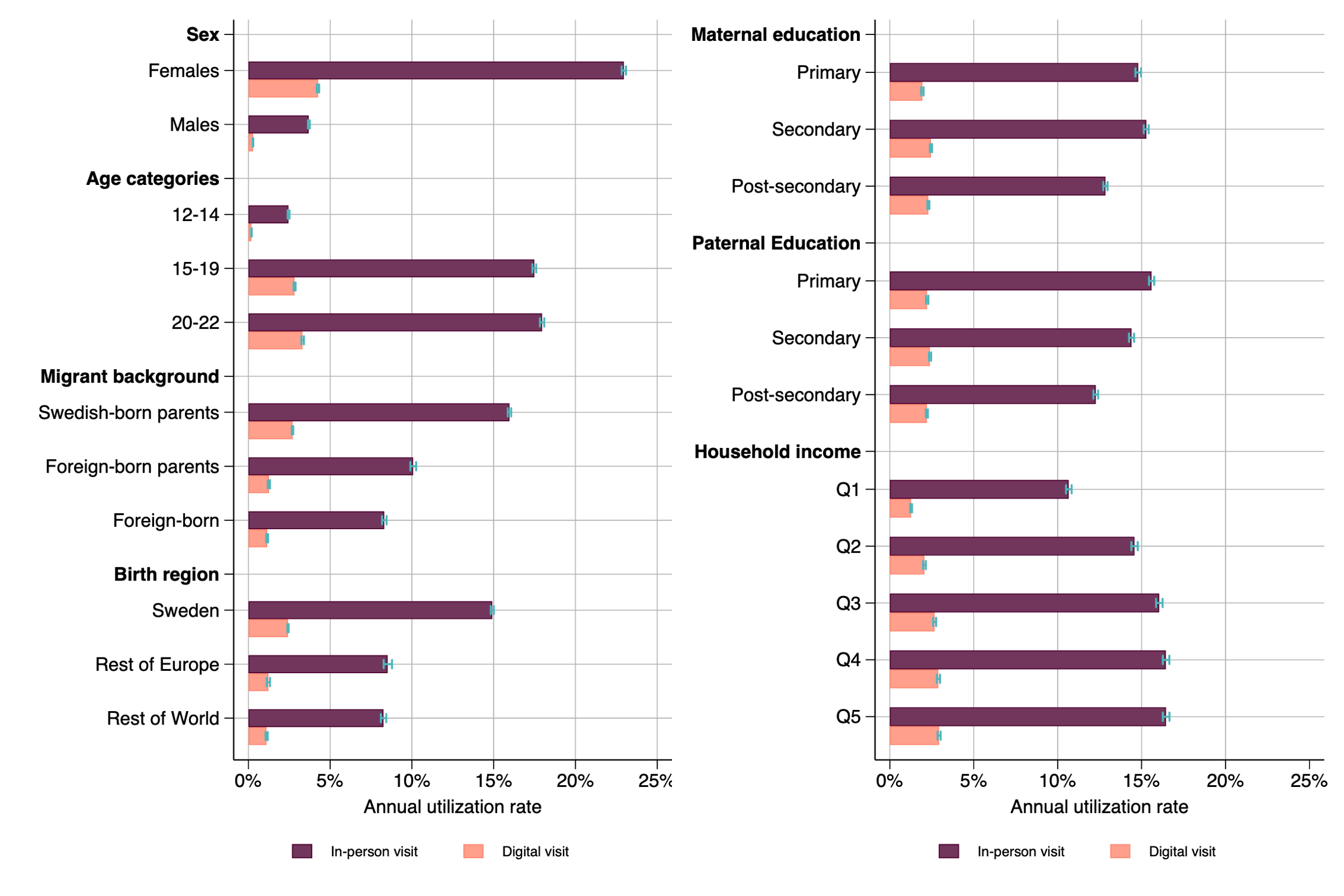


Supplemental Figure 4. Crude annual utilization rate and 95% confidence interval of in-person and digital Sexual and Reproductive Health (SRH) visits at population level across the study period (CI 95%). Error bars represent 95% confidence intervals.


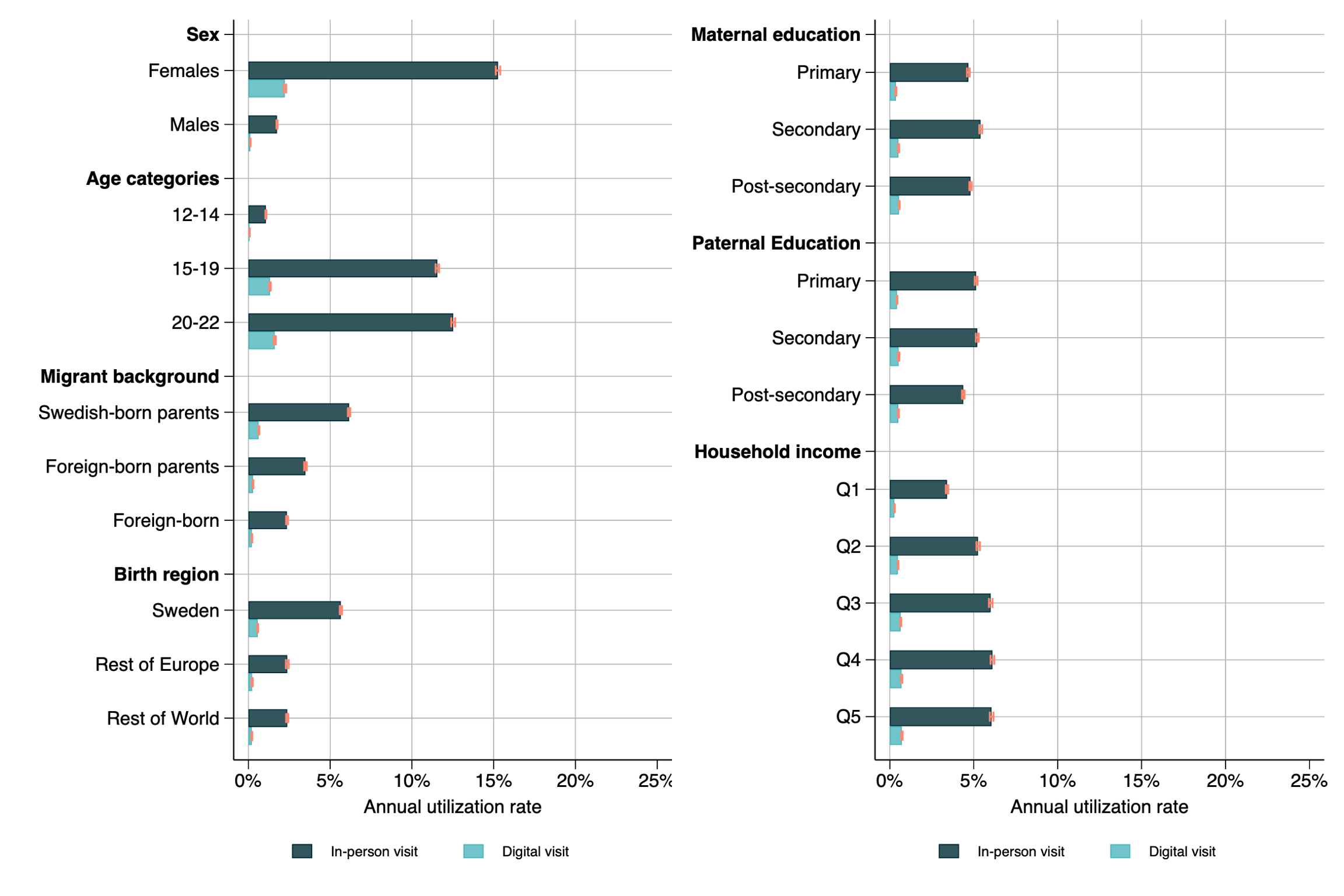


Supplemental Figure 5. Adjusted annual utilization rate and 95% confidence interval of in-person and digital Sexual and Reproductive Health (SRH) visits at population level across the study period (CI 95%). Estimates are sex and age-adjusted, except when analyzed as the strata. Error bars represent 95% confidence intervals.

**Supplemental Table 6.** **Annual utilization rate (%) of *in-person visits* among youth aged 12-22 in Stockholm County 2018-2022. Crude and age and sex-adjusted estimates, CI95%.**

|  |  |  |  |  |
| --- | --- | --- | --- | --- |
| In-person visits | **Crude estimates (%)** | **CI 95%** | **Adjusted estimates (%)** | **CI 95%** |
| Sex |  |  |  |  |
| Females | 22.96 | 22.83,23.09 | 15.27 | 15.13,15.40 |
| Males | 3.69 | 3.64,3.75 | 1.75 | 1.72,1.78 |
| Age category |  |  |  |  |
| 12-14 | 2.45 | 2.40,2.51 | 1.07 | 1.04,1.10 |
| 15-19 | 17.49 | 17.37,17.60 | 11.55 | 11.45,11.65 |
| 20-22 | 17.96 | 17.83,18.09 | 12.52 | 12.41,12.64 |
| Migrant background |  |  |  |  |
| Swedish-born parents | 15.97 | 15.86,16.07 | 6.16 | 6.09,6.23 |
| Foreign-born parents | 10.08 | 9.90,10.26 | 3.49 | 3.41,3.57 |
| Foreign-born | 8.31 | 8.17,8.45 | 2.36 | 2.31,2.41 |
| Birth region |  |  |  |  |
| Sweden | 14.91 | 14.82,15.01 | 5.65 | 5.59,5.72 |
| Rest of Europe | 8.52 | 8.27,8.78 | 2.37 | 2.29,2.46 |
| Rest of world | 8.26 | 8.10,8.43 | 2.37 | 2.31,2.43 |
| Maternal education level (2018-2020) |  |  |  |  |
| Primary education | 14.80 | 14.63,14.97 | 4.68 | 4.60,4.76 |
| Secondary education | 15.28 | 15.13,15.42 | 5.41 | 5.33,5.50 |
| Post-secondary education | 12.85 | 12.72,12.97 | 4.81 | 4.74,4.88 |
| Paternal education level (2018-2020) |  |  |  |  |
| Primary education | 15.59 | 15.44,15.75 | 5.14 | 5.06,5.22 |
| Secondary education | 14.40 | 14.25,14.55 | 5.21 | 5.13,5.29 |
| Post-secondary education | 12.27 | 12.13,12.41 | 4.37 | 4.30,4.45 |
| Household income (2018-2020) |  |  |  |  |
| Q1 | 10.66 | 10.50,10.83 | 3.41 | 3.34,3.48 |
| Q2 | 14.58 | 14.39,14.77 | 5.26 | 5.17,5.36 |
| Q3 | 16.05 | 15.86,16.25 | 6.01 | 5.91,6.12 |
| Q4 | 16.45 | 16.26,16.65 | 6.11 | 6.01,6.22 |
| Q5 | 16.46 | 16.26,16.66 | 6.06 | 5.96,6.17 |
|  |  |  |  |  |

Supplemental Table 7. Annual utilization rate (%) of *digital visits* among youth aged 12-22 in Stockholm County 2020-2022. Crude and age and sex-adjusted estimates, CI95%.

| Digital visits* | Crude  estimates (%) | CI 95% | Adjusted estimates  (%) | CI 95% |
| --- | --- | --- | --- | --- |
| Sex |  |  |  |  |
| Females | 4.26 | 4.19,4.32 | 2.23 | 2.16,2.30 |
| Males | 0.28 | 0.26,0.29 | 0.12 | 0.11,0.13 |
| Age category |  |  |  |  |
| 12-14 | 0.18 | 0.17,0.20 | 0.07 | 0.07,0.08 |
| 15-19 | 2.82 | 2.76,2.88 | 1.32 | 1.28,1.36 |
| 20-22 | 3.31 | 3.24,3.39 | 1.61 | 1.55,1.67 |
| Migrant background |  |  |  |  |
| Swedish-born parents | 2.70 | 2.65,2.74 | 0.63 | 0.61,0.66 |
| Foreign-born parents | 1.26 | 1.19,1.32 | 0.28 | 0.26,0.30 |
| Foreign-born | 1.15 | 1.09,1.20 | 0.22 | 0.21,0.23 |
| Birth region |  |  |  |  |
| Sweden | 2.42 | 2.38,2.46 | 0.57 | 0.54,0.59 |
| Rest of Europe | 1.22 | 1.12,1.32 | 0.23 | 0.21,0.26 |
| Rest of world | 1.11 | 1.05,1.18 | 0.21 | 0.20,0.23 |
| Maternal education level (2018-2020) |  |  |  |  |
| Primary education | 1.94 | 1.87,2.01 | 0.37 | 0.35,0.39 |
| Secondary education | 2.45 | 2.39,2.51 | 0.52 | 0.50,0.55 |
| Post-secondary education | 2.29 | 2.24,2.35 | 0.55 | 0.53,0.58 |
| Paternal education level (2018-2020) |  |  |  |  |
| Primary education | 2.22 | 2.16,2.29 | 0.43 | 0.41,0.45 |
| Secondary education | 2.39 | 2.33,2.46 | 0.53 | 0.50,0.56 |
| Post-secondary education | 2.21 | 2.15,2.27 | 0.51 | 0.49,0.54 |
| Household income (2018-2020) |  |  |  |  |
| Q1 | 1.27 | 1.21,1.32 | 0.27 | 0.25,0.29 |
| Q2 | 2.06 | 1.98,2.14 | 0.48 | 0.46,0.51 |
| Q3 | 2.67 | 2.58,2.76 | 0.65 | 0.62,0.68 |
| Q4 | 2.89 | 2.80,2.99 | 0.70 | 0.67,0.74 |
| Q5 | 2.94 | 2.85,3.03 | 0.72 | 0.68,0.76 |
|  |  |  |  |  |

*Digital visits include video and chat consultations from 2020 and onwards.

Supplemental Table 8. Annual utilization rate (%) of *video consultations* among youth aged 12-22 in Stockholm County 2020-2022. Crude and age and sex-adjusted estimates, CI95%.

| Video consultations | Crude  estimates (%) | CI 95% | Adjusted estimates  (%) | CI 95% |
| --- | --- | --- | --- | --- |
| Sex |  |  |  |  |
| Females | 3.31 | 3.25,3.37 | 1.62 | 1.57,1.69 |
| Males | 0.07 | 0.07,0.08 | 0.03 | 0.03,0.03 |
| Age category |  |  |  |  |
| 12-14 | 0.11 | 0.10,0.12 | 0.03 | 0.02,0.03 |
| 15-19 | 2.12 | 2.07,2.17 | 0.60 | 0.57,0.64 |
| 20-22 | 2.41 | 2.35,2.48 | 0.71 | 0.67,0.76 |
| Migrant background |  |  |  |  |
| Swedish-born parents | 2.06 | 2.02,2.10 | 0.27 | 0.26,0.29 |
| Foreign-born parents | 0.77 | 0.72,0.82 | 0.09 | 0.08,0.10 |
| Foreign-born | 0.75 | 0.71,0.79 | 0.08 | 0.07,0.08 |
| Birth region |  |  |  |  |
| Sweden | 1.81 | 1.77,1.84 | 0.24 | 0.22,0.25 |
| Rest of Europe | 0.84 | 0.76,0.93 | 0.09 | 0.08,0.10 |
| Rest of world | 0.71 | 0.66,0.76 | 0.07 | 0.07,0.08 |
| Maternal education level (2018-2020) |  |  |  |  |
| Primary education | 1.34 | 1.29,1.40 | 0.14 | 0.13,0.15 |
| Secondary education | 1.84 | 1.78,1.89 | 0.22 | 0.21,0.24 |
| Post-secondary education | 1.73 | 1.68,1.78 | 0.24 | 0.22,0.25 |
| Paternal education level (2018-2020) |  |  |  |  |
| Primary education | 1.61 | 1.56,1.67 | 0.18 | 0.16,0.19 |
| Secondary education | 1.78 | 1.72,1.83 | 0.22 | 0.21,0.24 |
| Post-secondary education | 1.67 | 1.62,1.73 | 0.22 | 0.21,0.24 |
| Household income (2018-2020) |  |  |  |  |
| Q1 | 0.80 | 0.75,0.84 | 0.27 | 0.25,0.29 |
| Q2 | 1.44 | 1.38,1.51 | 0.48 | 0.46,0.51 |
| Q3 | 1.98 | 1.91,2.06 | 0.65 | 0.62,0.68 |
| Q4 | 2.25 | 2.17,2.33 | 0.70 | 0.67,0.74 |
| Q5 | 2.31 | 2.23,2.40 | 0.72 | 0.68,0.76 |
|  |  |  |  |  |

Supplemental Table 9. Annual utilization rate (%) of *chat consultations* among youth aged 12-22 in Stockholm County 2020-2022. Crude and age and sex-adjusted estimates, CI95%.

| Chat consultations | Crude  estimates (%) | CI 95% | Adjusted estimates  (%) | CI 95% |
| --- | --- | --- | --- | --- |
| Sex |  |  |  |  |
| Females | 1.36 | 1.32,1.39 | 0.80 | 0.77,0.84 |
| Males | 0.21 | 0.20,0.22 | 0.12 | 0.11,0.13 |
| Age category |  |  |  |  |
| 12-14 | 0.08 | 0.07,0.09 | 0.05 | 0.05,0.06 |
| 15-19 | 0.98 | 0.95,1.01 | 0.66 | 0.63,0.69 |
| 20-22 | 1.14 | 1.10,1.18 | 0.79 | 0.75,0.82 |
| Migrant background |  |  |  |  |
| Swedish-born parents | 0.87 | 0.85,0.90 | 0.35 | 0.33,0.36 |
| Foreign-born parents | 0.59 | 0.55,0.63 | 0.24 | 0.22,0.26 |
| Foreign-born | 0.50 | 0.47,0.54 | 0.18 | 0.16,0.19 |
| Birth region |  |  |  |  |
| Sweden | 0.82 | 0.80,0.84 | 0.32 | 0.31,0.34 |
| Rest of Europe | 0.46 | 0.41,0.53 | 0.16 | 0.14,0.19 |
| Rest of world | 0.52 | 0.48,0.56 | 0.18 | 0.17,0.20 |
| Maternal education level (2018-2020) |  |  |  |  |
| Primary education | 0.76 | 0.72,0.80 | 0.26 | 0.24,0.28 |
| Secondary education | 0.83 | 0.80,0.86 | 0.31 | 0.29,0.33 |
| Post-secondary education | 0.76 | 0.73,0.79 | 0.31 | 0.29,0.33 |
| Paternal education level (2018-2020) |  |  |  |  |
| Primary education | 0.79 | 0.76,0.83 | 0.27 | 0.25,0.29 |
| Secondary education | 0.83 | 0.79,0.86 | 0.32 | 0.30,0.34 |
| Post-secondary education | 0.73 | 0.70,0.77 | 0.29 | 0.27,0.31 |
| Household income (2018-2020) |  |  |  |  |
| Q1 | 0.57 | 0.54,0.61 | 0.22 | 0.21,0.24 |
| Q2 | 0.79 | 0.75,0.84 | 0.32 | 0.30,0.35 |
| Q3 | 0.90 | 0.86,0.95 | 0.37 | 0.35,0.40 |
| Q4 | 0.88 | 0.84,0.93 | 0.36 | 0.34,0.39 |
| Q5 | 0.89 | 0.85,0.94 | 0.36 | 0.34,0.39 |
|  |  |  |  |  |

Supplemental Table 10. Sex-stratified Annual utilization rate (%) of *in-person visits* among youth aged 12-22 in Stockholm County 2018-2022. Crude and age and age-adjusted estimates, CI95%.

|  | Females | | | | Males | | | |
| --- | --- | --- | --- | --- | --- | --- | --- | --- |
| In-person visits | **Crude**  **estimates (%)** | **CI 95%** | **Adjusted estimates**  **(%)** | **CI 95%** | **Crude**  **estimates (%)** | **CI 95%** | **Adjusted estimates**  **(%)** | **CI 95%** |
| Annual utilization rate | 22.99 | 22.86,23.12 | 15.20 | 15.06,15.35 | 3.64 | 3.6,3.7 | 1.73 | 1.68,1.78 |
| Age category |  |  |  |  |  |  |  |  |
| 12-14 | 4.30 | 4.20,4.40 | 4.30 | 4.20,4.40 | 0.29 | 0.3,0.3 | 0.29 | 0.27,0.31 |
| 15-19 | 30.87 | 30.68,31.07 | 30.87 | 30.68,31.07 | 3.97 | 3.9,4.0 | 3.97 | 3.90,4.05 |
| 20-22 | 30.53 | 30.31,30.76 | 30.53 | 30.31,30.76 | 6.25 | 6.1,6.4 | 6.25 | 6.14,6.36 |
| Migrant background |  |  |  |  |  |  |  |  |
| Swedish-born parents | 27.54 | 27.37,27.71 | 18.87 | 18.69,19.05 | 3.71 | 3.6,3.8 | 1.82 | 1.77,1.88 |
| Foreign-born parents | 15.40 | 15.12,15.69 | 9.52 | 9.32,9.74 | 3.86 | 3.7,4.0 | 1.98 | 1.89,2.07 |
| Foreign-born | 13.32 | 13.08,13.56 | 6.77 | 6.63,6.92 | 3.26 | 3.2,3.4 | 1.26 | 1.20,1.31 |
| Birth region |  |  |  |  |  |  |  |  |
| Sweden | 25.28 | 25.13,25.43 | 17.14 | 16.98,17.30 | 3.74 | 3.7,3.8 | 1.85 | 1.80,1.91 |
| Rest of Europe | 14.17 | 13.76,14.60 | 7.59 | 7.34,7.86 | 2.44 | 2.3,2.6 | 1.03 | 0.95,1.11 |
| Rest of world | 12.93 | 12.65,13.22 | 6.47 | 6.30,6.64 | 3.59 | 3.5,3.7 | 1.35 | 1.28,1.41 |
| Maternal education level (2018-2020) |  |  |  |  |  |  |  |  |
| Primary education | 24.04 | 23.77,24.31 | 14.07 | 13.85,14.28 | 4.47 | 4.4,4.6 | 1.81 | 1.74,1.88 |
| Secondary education | 25.83 | 25.59,26.07 | 16.93 | 16.72,17.15 | 3.75 | 3.7,3.8 | 1.69 | 1.63,1.76 |
| Post-secondary education | 21.82 | 21.62,22.03 | 15.11 | 14.92,15.30 | 3.13 | 3.1,3.2 | 1.59 | 1.53,1.65 |
| Paternal education level (2018-2020) |  |  |  |  |  |  |  |  |
| Primary education | 26.01 | 25.76,26.25 | 15.90 | 15.69,16.12 | 4.27 | 4.2,4.4 | 1.77 | 1.70,1.83 |
| Secondary education | 24.43 | 24.19,24.66 | 16.43 | 16.21,16.64 | 3.53 | 3.4,3.6 | 1.66 | 1.60,1.73 |
| Post-secondary education | 20.69 | 20.46,20.92 | 13.83 | 13.63,14.03 | 3.10 | 3.0,3.2 | 1.54 | 1.48,1.60 |
| Household income (2018-2020) |  |  |  |  |  |  |  |  |
| Q1 | 16.76 | 16.50,17.02 | 10.06 | 9.87,10.25 | 3.55 | 3.4,3.7 | 1.64 | 1.58,1.72 |
| Q2 | 24.38 | 24.07,24.69 | 16.14 | 15.88,16.40 | 3.80 | 3.7,3.9 | 1.80 | 1.73,1.88 |
| Q3 | 27.30 | 26.98,27.63 | 18.64 | 18.35,18.92 | 3.83 | 3.7,4.0 | 1.82 | 1.74,1.90 |
| Q4 | 27.95 | 27.62,28.28 | 19.00 | 18.72,19.29 | 3.81 | 3.7,3.9 | 1.80 | 1.73,1.88 |
| Q5 | 27.35 | 27.03,27.68 | 18.41 | 18.14,18.69 | 4.18 | 4.1,4.3 | 1.98 | 1.90,2.06 |

Supplemental Table 11. Sex-stratified Annual utilization rate (%) of *digital visits* among youth aged 12-22 in Stockholm County 2020-2022. Crude and age and age-adjusted estimates, CI95%.

|  | Females | | | | Males | | | |
| --- | --- | --- | --- | --- | --- | --- | --- | --- |
| Digital visits* | **Crude**  **estimates (%)** | **CI 95%** | **Adjusted estimates**  **(%)** | **CI 95%** | **Crude**  **estimates (%)** | **CI 95%** | **Adjusted estimates**  **(%)** | **CI 95%** |
| Annual utilization rate | 4.26 | 4.19,4.32 | 2.15 | 2.08,2.22 | 0.28 | 0.26,0.29 | 0.17 | 0.16,0.19 |
| Age category |  |  |  |  |  |  |  |  |
| 12-14 | 0.33 | 0.30,0.36 | 0.33 | 0.30,0.36 | 0.04 | 0.03,0.05 | 0.04 | 0.03,0.05 |
| 15-19 | 5.54 | 5.43,5.65 | 5.54 | 5.43,5.65 | 0.29 | 0.27,0.31 | 0.29 | 0.27,0.31 |
| 20-22 | 6.41 | 6.26,6.57 | 6.41 | 6.26,6.57 | 0.51 | 0.47,0.55 | 0.51 | 0.47,0.55 |
| Migrant background |  |  |  |  |  |  |  |  |
| Swedish-born parents | 5.28 | 5.19,5.38 | 2.72 | 2.63,2.81 | 0.28 | 0.27,0.30 | 0.18 | 0.17,0.20 |
| Foreign-born parents | 2.31 | 2.19,2.44 | 1.16 | 1.09,1.23 | 0.28 | 0.25,0.33 | 0.19 | 0.16,0.22 |
| Foreign-born | 2.23 | 2.12,2.34 | 0.93 | 0.87,0.98 | 0.25 | 0.22,0.28 | 0.13 | 0.11,0.15 |
| Birth region |  |  |  |  |  |  |  |  |
| Sweden | 4.72 | 4.64,4.80 | 2.42 | 2.35,2.50 | 0.28 | 0.27,0.30 | 0.18 | 0.17,0.20 |
| Rest of Europe | 2.30 | 2.12,2.51 | 0.99 | 0.90,1.09 | 0.23 | 0.18,0.29 | 0.13 | 0.10,0.17 |
| Rest of world | 2.19 | 2.06,2.33 | 0.89 | 0.83,0.96 | 0.25 | 0.22,0.30 | 0.13 | 0.11,0.15 |
| Maternal education level (2018-2020) |  |  |  |  |  |  |  |  |
| Primary education | 3.70 | 3.58,3.84 | 1.60 | 1.52,1.68 | 0.30 | 0.26,0.33 | 0.16 | 0.14,0.18 |
| Secondary education | 4.79 | 4.67,4.92 | 2.32 | 2.23,2.41 | 0.26 | 0.24,0.29 | 0.15 | 0.14,0.18 |
| Post-secondary education | 4.47 | 4.37,4.58 | 2.40 | 2.32,2.50 | 0.28 | 0.25,0.30 | 0.18 | 0.16,0.21 |
| Paternal education level (2018-2020) |  |  |  |  |  |  |  |  |
| Primary education | 4.31 | 4.18,4.43 | 1.91 | 1.83,1.99 | 0.30 | 0.27,0.33 | 0.16 | 0.14,0.18 |
| Secondary education | 4.68 | 4.56,4.81 | 2.35 | 2.26,2.45 | 0.27 | 0.25,0.30 | 0.17 | 0.15,0.19 |
| Post-secondary education | 4.32 | 4.20,4.45 | 2.26 | 2.17,2.36 | 0.26 | 0.24,0.29 | 0.17 | 0.15,0.19 |
| Household income (2018-2020) |  |  |  |  |  |  |  |  |
| Q1 | 2.39 | 2.28,2.51 | 1.18 | 1.11,1.24 | 0.23 | 0.20,0.26 | 0.14 | 0.12,0.17 |
| Q2 | 4.01 | 3.86,4.17 | 2.11 | 2.01,2.21 | 0.28 | 0.24,0.31 | 0.18 | 0.15,0.21 |
| Q3 | 5.21 | 5.04,5.39 | 2.80 | 2.68,2.93 | 0.32 | 0.28,0.36 | 0.21 | 0.18,0.24 |
| Q4 | 5.63 | 5.45,5.82 | 3.04 | 2.91,3.17 | 0.31 | 0.27,0.35 | 0.20 | 0.17,0.23 |
| Q5 | 5.72 | 5.54,5.91 | 3.12 | 2.99,3.26 | 0.30 | 0.27,0.35 | 0.20 | 0.17,0.23 |
|  |  |  |  |  |  |  |  |  |

*Digital visits include video and chat consultations data from 2020 and onwards.

Supplemental Table 12. Sex-stratified Annual utilization rate (%) of *video consultations* among youth aged 12-22 in Stockholm County 2020-2022. Crude and age and age-adjusted estimates, CI95%.

|  | Females | | | | Males | | | |
| --- | --- | --- | --- | --- | --- | --- | --- | --- |
| Video consultations | **Crude**  **estimates (%)** | **CI 95%** | **Adjusted estimates**  **(%)** | **CI 95%** | **Crude**  **estimates (%)** | **CI 95%** | **Adjusted estimates**  **(%)** | **CI 95%** |
| Annual utilization rate | 3.31 | 3.25,3.37 | 1.60 | 1.54,1.66 | 0.08 | 0.07,0.08 | 0.04 | 0.03,0.05 |
| Age category |  |  |  |  |  |  |  |  |
| 12-14 | 0.22 | 0.19,0.25 | 0.22 | 0.19,0.25 | 0.01 | 0.01,0.02 | 0.01 | 0.01,0.02 |
| 15-19 | 4.36 | 4.26,4.46 | 4.36 | 4.26,4.46 | 0.06 | 0.05,0.08 | 0.06 | 0.05,0.08 |
| 20-22 | 4.94 | 4.81,5.08 | 4.94 | 4.81,5.08 | 0.16 | 0.14,0.18 | 0.16 | 0.14,0.18 |
| Migrant background |  |  |  |  |  |  |  |  |
| Swedish-born parents | 4.22 | 4.14,4.30 | 2.08 | 2.00,2.16 | 0.08 | 0.07,0.09 | 0.04 | 0.04,0.05 |
| Foreign-born parents | 1.55 | 1.45,1.65 | 0.73 | 0.68,0.79 | 0.07 | 0.06,0.09 | 0.04 | 0.03,0.05 |
| Foreign-born | 1.57 | 1.48,1.67 | 0.62 | 0.58,0.67 | 0.07 | 0.07,0.09 | 0.03 | 0.02,0.04 |
| Birth region |  |  |  |  |  |  |  |  |
| Sweden | 3.71 | 3.64,3.78 | 1.82 | 1.76,1.89 | 0.08 | 0.07,0.09 | 0.08 | 0.07,0.09 |
| Rest of Europe | 1.71 | 1.55,1.88 | 0.70 | 0.63,0.79 | 0.07 | 0.06,0.09 | 0.07 | 0.06,0.09 |
| Rest of world | 1.51 | 1.40,1.62 | 0.58 | 0.54,0.64 | 0.08 | 0.07,0.09 | 0.08 | 0.07,0.09 |
| Maternal education level (2018-2020) |  |  |  |  |  |  |  |  |
| Primary education | 2.72 | 2.61,2.83 | 1.12 | 1.06,1.18 | 0.08 | 0.07,0.10 | 0.04 | 0.03,0.05 |
| Secondary education | 3.77 | 3.66,3.88 | 1.74 | 1.66,1.82 | 0.08 | 0.07,0.09 | 0.04 | 0.03,0.05 |
| Post-secondary education | 3.56 | 3.46,3.66 | 1.83 | 1.75,1.91 | 0.08 | 0.07,0.09 | 0.04 | 0.03,0.05 |
| Paternal education level (2018-2020) |  |  |  |  |  |  |  |  |
| Primary education | 3.29 | 3.19,3.40 | 1.39 | 1.32,1.46 | 0.08 | 0.07,0.10 | 0.04 | 0.03,0.05 |
| Secondary education | 3.66 | 3.55,3.77 | 1.75 | 1.67,1.84 | 0.08 | 0.07,0.09 | 0.04 | 0.03,0.05 |
| Post-secondary education | 3.44 | 3.33,3.56 | 1.73 | 1.64,1.81 | 0.08 | 0.07,0.09 | 0.04 | 0.03,0.05 |
| Household income (2018-2020) |  |  |  |  |  |  |  |  |
| Q1 | 1.61 | 1.52,1.70 | 0.75 | 0.70,0.81 | 0.06 | 0.05,0.08 | 0.03 | 0.02,0.04 |
| Q2 | 2.99 | 2.86,3.13 | 1.50 | 1.42,1.59 | 0.06 | 0.05,0.08 | 0.03 | 0.02,0.05 |
| Q3 | 4.07 | 3.92,4.23 | 2.10 | 2.00,2.21 | 0.09 | 0.07,0.11 | 0.05 | 0.03,0.06 |
| Q4 | 4.58 | 4.41,4.75 | 2.38 | 2.27,2.50 | 0.09 | 0.07,0.11 | 0.05 | 0.04,0.06 |
| Q5 | 4.70 | 4.54,4.88 | 2.48 | 2.36,2.60 | 0.08 | 0.07,0.11 | 0.05 | 0.03,0.06 |
|  |  |  |  |  |  |  |  |  |

Supplemental Table 13. Sex-stratified Annual utilization rate (%) of chat consultations among youth aged 12-22 in Stockholm County 2020-2022. Crude and age and age-adjusted estimates, CI95%.

|  | Females | | | | Males | | | |
| --- | --- | --- | --- | --- | --- | --- | --- | --- |
| Chat consultations | **Crude**  **estimates (%)** | **CI 95%** | **Adjusted estimates**  **(%)** | **CI 95%** | **Crude**  **estimates (%)** | **CI 95%** | **Adjusted estimates**  **(%)** | **CI 95%** |
| Annual utilization rate | 1.36 | 1.32,1.39 | 0.77 | 0.73,0.81 | 0.21 | 0.20,0.23 | 0.14 | 0.13,0.15 |
| Age category |  |  |  |  |  |  |  |  |
| 12-14 | 0.13 | 0.11,0.15 | 0.13 | 0.11,0.15 | 0.04 | 0.03,0.05 | 0.04 | 0.03,0.05 |
| 15-19 | 1.78 | 1.72,1.84 | 1.78 | 1.72,1.84 | 0.24 | 0.22,0.26 | 0.24 | 0.22,0.26 |
| 20-22 | 2.01 | 1.93,2.10 | 2.01 | 1.93,2.10 | 0.36 | 0.33,0.40 | 0.36 | 0.33,0.40 |
| Migrant background |  |  |  |  |  |  |  |  |
| Swedish-born parents | 1.58 | 1.54,1.63 | 0.91 | 0.87,0.96 | 0.22 | 0.20,0.23 | 0.14 | 0.13,0.16 |
| Foreign-born parents | 0.98 | 0.91,1.06 | 0.58 | 0.53,0.63 | 0.23 | 0.20,0.27 | 0.16 | 0.13,0.19 |
| Foreign-born | 0.87 | 0.80,0.94 | 0.43 | 0.40,0.47 | 0.19 | 0.17,0.22 | 0.11 | 0.09,0.13 |
| Birth region |  |  |  |  |  |  |  |  |
| Sweden | 1.47 | 1.43,1.51 | 0.85 | 0.81,0.89 | 0.22 | 0.20,0.23 | 0.15 | 0.13,0.16 |
| Rest of Europe | 0.76 | 0.66,0.88 | 0.39 | 0.34,0.46 | 0.18 | 0.14,0.24 | 0.11 | 0.08,0.15 |
| Rest of world | 0.92 | 0.84,1.00 | 0.45 | 0.41,0.50 | 0.20 | 0.16,0.23 | 0.10 | 0.09,0.13 |
| Maternal education level (2018-2020) |  |  |  |  |  |  |  |  |
| Primary education | 1.33 | 1.26,1.41 | 0.67 | 0.63,0.72 | 0.22 | 0.19,0.25 | 0.12 | 0.10,0.14 |
| Secondary education | 1.51 | 1.44,1.57 | 0.83 | 0.78,0.88 | 0.20 | 0.18,0.22 | 0.12 | 0.11,0.14 |
| Post-secondary education | 1.34 | 1.28,1.39 | 0.81 | 0.76,0.86 | 0.22 | 0.20,0.24 | 0.15 | 0.13,0.17 |
| Paternal education level (2018-2020) |  |  |  |  |  |  |  |  |
| Primary education | 1.41 | 1.34,1.48 | 0.72 | 0.68,0.77 | 0.22 | 0.19,0.24 | 0.12 | 0.10,0.14 |
| Secondary education | 1.49 | 1.43,1.56 | 0.85 | 0.80,0.90 | 0.21 | 0.19,0.24 | 0.13 | 0.12,0.15 |
| Post-secondary education | 1.30 | 1.24,1.37 | 0.77 | 0.72,0.82 | 0.21 | 0.18,0.23 | 0.14 | 0.12,0.16 |
| Household income (2018-2020) |  |  |  |  |  |  |  |  |
| Q1 | 0.99 | 0.93,1.06 | 0.57 | 0.53,0.62 | 0.18 | 0.16,0.21 | 0.12 | 0.10,0.14 |
| Q2 | 1.42 | 1.34,1.51 | 0.85 | 0.79,0.91 | 0.22 | 0.19,0.26 | 0.15 | 0.13,0.18 |
| Q3 | 1.62 | 1.53,1.71 | 0.97 | 0.91,1.04 | 0.24 | 0.21,0.28 | 0.17 | 0.14,0.20 |
| Q4 | 1.58 | 1.49,1.67 | 0.95 | 0.89,1.02 | 0.23 | 0.20,0.27 | 0.16 | 0.14,0.19 |
| Q5 | 1.60 | 1.51,1.70 | 0.97 | 0.90,1.04 | 0.23 | 0.20,0.26 | 0.16 | 0.13,0.18 |
|  |  |  |  |  |  |  |  |  |


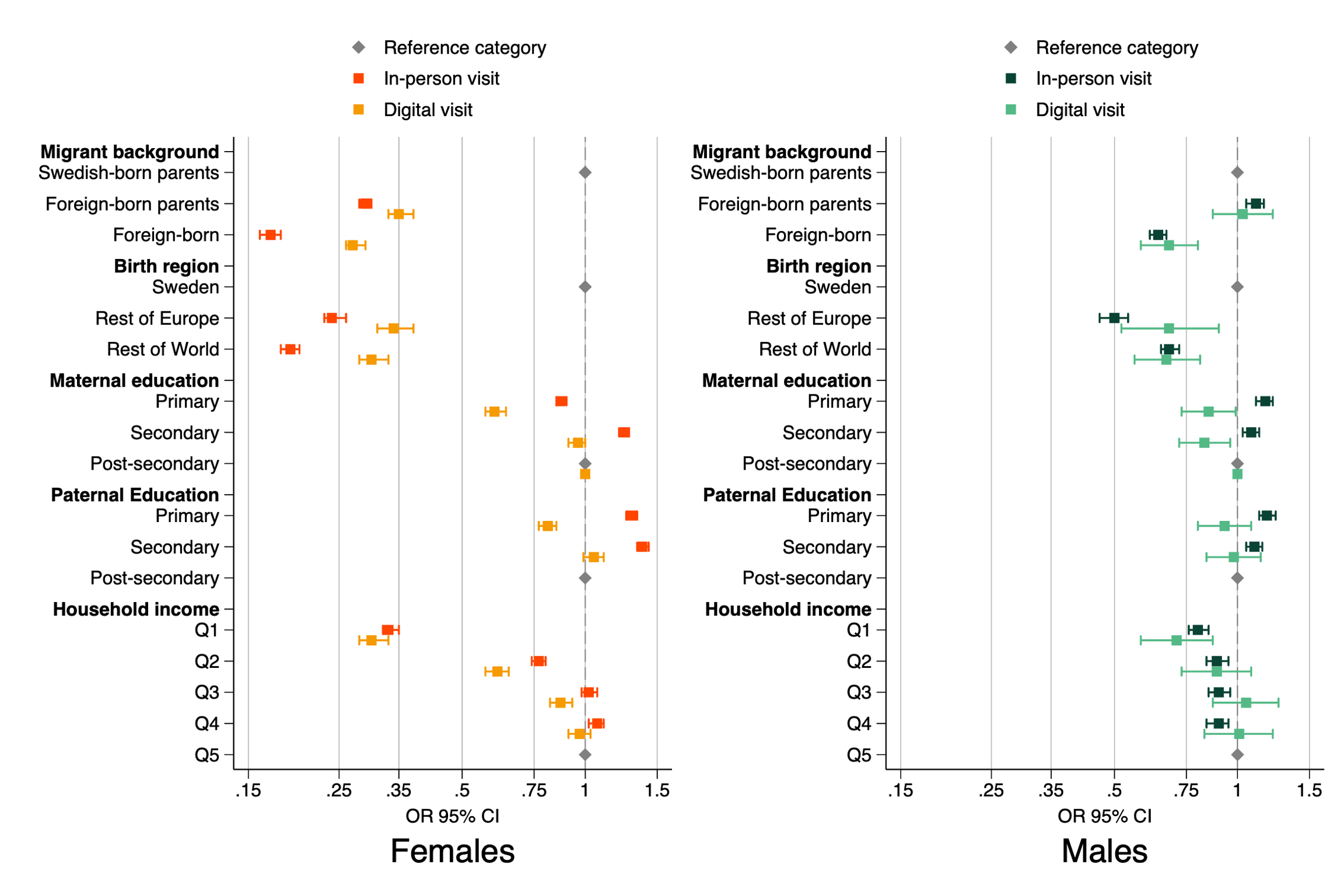


Supplemental Figure 6. Sex-stratified Odds Ratios and 95% confidence interval of SRH-contacts at population level for in-person and digital visits across the entire study period 2018-2022. Estimates are age adjusted. Error bars represent 95% confidence intervals.

Supplemental Table 14. Odds Ratios and 95% confidence interval of SRH-use at population level for *in-person visits*, 2018-2022 (CI 95%). Crude and age and sex-adjusted estimates.

| In-person visits 2018-2022 | Crude OR | CI 95% | Adjusted OR | CI 95% |
| --- | --- | --- | --- | --- |
| Sex |  |  |  |  |
| Females | 1.00 | 1.00,1.00 | 1.00 | 1.00,1.00 |
| Males | 0.05 | 0.05,0.05 | 0.04 | 0.04,0.04 |
| Age category |  |  |  |  |
| 12-14 | 0.04 | 0.04,0.04 | 0.04 | 0.04,0.04 |
| 15-19 | 1.00 | 1.00,1.00 | 1.00 | 1.00,1.00 |
| 20-22 | 1.06 | 1.04,1.08 | 1.15 | 1.13,1.16 |
| Migrant background |  |  |  |  |
| Swedish-born parents | 1.00 | 1.00,1.00 | 1.00 | 1.00,1.00 |
| Foreign-born parents | 0.42 | 0.40,0.43 | 0.44 | 0.42,0.45 |
| Foreign-born | 0.30 | 0.29,0.31 | 0.26 | 0.25,0.26 |
| Birth region |  |  |  |  |
| Sweden | 1.00 | 1.00,1.00 | 1.00 | 1.00,1.00 |
| Rest of Europe | 0.36 | 0.34,0.38 | 0.29 | 0.28,0.31 |
| Rest of world | 0.34 | 0.33,0.35 | 0.29 | 0.28,0.30 |
| Maternal education level (2018-2020) |  |  |  |  |
| Primary education | 1.32 | 1.28,1.36 | 0.96 | 0.93,0.99 |
| Secondary education | 1.41 | 1.37,1.44 | 1.19 | 1.17,1.22 |
| Post-secondary education | 1.00 | 1.00,1.00 | 1.00 | 1.00,1.00 |
| Paternal education level (2018-2020) |  |  |  |  |
| Primary education | 1.60 | 1.55,1.64 | 1.27 | 1.24,1.30 |
| Secondary education | 1.36 | 1.32,1.40 | 1.29 | 1.26,1.33 |
| Post-secondary education | 1.00 | 1.00,1.00 | 1.00 | 1.00,1.00 |
| Household income (2018-2020) |  |  |  |  |
| Q1 | 0.43 | 0.42,0.45 | 0.43 | 0.42,0.45 |
| Q2 | 0.78 | 0.75,0.81 | 0.81 | 0.78,0.83 |
| Q3 | 0.95 | 0.92,0.98 | 0.99 | 0.96,1.02 |
| Q4 | 1.00 | 0.96,1.04 | 1.01 | 0.98,1.05 |
| Q5 | 1.00 | 1.00,1.00 | 1.00 | 1.00,1.00 |
|  |  |  |  |  |

.

Supplemental Table 15. Odds Ratios and 95% confidence interval of SRH-use at population level for *digital visits*, 2020-2022 (CI 95%). Crude and age and sex-adjusted estimates.

| Digital visits | Crude OR | CI 95% | Adjusted OR | CI 95% |
| --- | --- | --- | --- | --- |
| Sex |  |  |  |  |
| Females | 1.00 | 1.00,1.00 | 1.00 | 1.00,1.00 |
| Males | 0.04 | 0.04,0.05 | 0.04 | 0.04,0.04 |
| Age category |  |  |  |  |
| 12-14 | 0.04 | 0.04,0.05 | 0.04 | 0.04,0.05 |
| 15-19 | 1.00 | 1.00,1.00 | 1.00 | 1.00,1.00 |
| 20-22 | 1.23 | 1.19,1.28 | 1.27 | 1.22,1.31 |
| Migrant background |  |  |  |  |
| Swedish-born parents | 1.00 | 1.00,1.00 | 1.00 | 1.00,1.00 |
| Foreign-born parents | 0.39 | 0.37,0.42 | 0.41 | 0.38,0.43 |
| Foreign-born | 0.35 | 0.33,0.37 | 0.31 | 0.29,0.33 |
| Birth region |  |  |  |  |
| Sweden | 1.00 | 1.00,1.00 | 1.00 | 1.00,1.00 |
| Rest of Europe | 0.43 | 0.39,0.48 | 0.37 | 0.33,0.41 |
| Rest of world | 0.39 | 0.36,0.42 | 0.34 | 0.32,0.37 |
| Maternal education level (2018-2020) |  |  |  |  |
| Primary education | 0.81 | 0.77,0.86 | 0.63 | 0.60,0.66 |
| Secondary education | 1.09 | 1.04,1.14 | 0.94 | 0.90,0.99 |
| Post-secondary education | 1.00 | 1.00,1.00 | 1.00 | 1.00,1.00 |
| Paternal education level (2018-2020) |  |  |  |  |
| Primary education | 1.01 | 0.96,1.06 | 0.82 | 0.78,0.87 |
| Secondary education | 1.11 | 1.06,1.16 | 1.04 | 0.99,1.09 |
| Post-secondary education | 1.00 | 1.00,1.00 | 1.00 | 1.00,1.00 |
| Household income (2018-2020) |  |  |  |  |
| Q1 | 0.35 | 0.33,0.38 | 0.34 | 0.31,0.36 |
| Q2 | 0.63 | 0.60,0.68 | 0.64 | 0.60,0.68 |
| Q3 | 0.88 | 0.83,0.94 | 0.89 | 0.84,0.94 |
| Q4 | 0.98 | 0.92,1.04 | 0.97 | 0.92,1.03 |
| Q5 | 1.00 | 1.00,1.00 | 1.00 | 1.00,1.00 |
|  |  |  |  |  |

*Digital visits include video and chat consultations from 2020 and onwards. .

Supplemental Table 16. Sex-stratified Odds Ratios of *in-person visits* and *digital visits* among youth aged 12-22 in Stockholm County 2018-2022. Crude estimates, CI95%.

|  | In-person visits | | | | Digital visits* | | | |
| --- | --- | --- | --- | --- | --- | --- | --- | --- |
|  | **Females** | | **Males** | | **Females** | | **Males** | |
|  | **Crude**  **OR** | **CI 95%** | **Crude**  **OR** | **CI 95%** | **Crude OR** | **CI 95%** | **Crude**  **OR** | **CI 95%** |
| Age category |  |  |  |  |  |  |  |  |
| 12-14 | 0.04 | 0.04,0.04 | 0.05 | 0.05,0.06 | 0.04 | 0.04,0.04 | 0.14 | 0.10,0.18 |
| 15-19 | 1.00 | 1.00,1.00 | 1.00 | 1.00,1.00 | 1.00 | 1.00,1.00 | 1.00 | 1.00,1.00 |
| 20-22 | 0.97 | 0.96,0.99 | 1.80 | 1.74,1.85 | 1.22 | 1.17,1.27 | 1.80 | 1.60,2.03 |
| Migrant background |  |  |  |  |  |  |  |  |
| Swedish-born parents | 1.00 | 1.00,1.00 | 1.00 | 1.00,1.00 | 1.00 | 1.00,1.00 | 1.00 | 1.00,1.00 |
| Foreign-born parents | 0.31 | 0.30,0.33 | 1.05 | 1.00,1.11 | 0.35 | 0.33,0.38 | 0.99 | 0.84,1.17 |
| Foreign-born | 0.24 | 0.24,0.25 | 0.84 | 0.80,0.88 | 0.34 | 0.31,0.36 | 0.85 | 0.73,1.00 |
| Birth region |  |  |  |  |  |  |  |  |
| Sweden | 1.00 | 1.00,1.00 | 1.00 | 1.00,1.00 | 1.00 | 1.00,1.00 | 1.00 | 1.00,1.00 |
| Rest of Europe | 0.32 | 0.31,0.34 | 0.58 | 0.53,0.63 | 0.40 | 0.36,0.45 | 0.78 | 0.60,1.03 |
| Rest of world | 0.28 | 0.27,0.29 | 0.95 | 0.90,1.00 | 0.38 | 0.35,0.41 | 0.89 | 0.74,1.06 |
| Maternal education level (2018-2020) |  |  |  |  |  |  |  |  |
| Primary education | 1.23 | 1.19,1.27 | 1.60 | 1.52,1.68 | 0.78 | 0.74,0.83 | 1.07 | 0.92,1.25 |
| Secondary education | 1.44 | 1.40,1.48 | 1.27 | 1.21,1.33 | 1.10 | 1.05,1.15 | 0.94 | 0.82,1.08 |
| Post-secondary education | 1.00 | 1.00,1.00 | 1.00 | 1.00,1.00 | 1.00 | 1.00,1.00 | 1.00 | 1.00,1.00 |
| Paternal education level (2018-2020) |  |  |  |  |  |  |  |  |
| Primary education | 1.63 | 1.58,1.68 | 1.52 | 1.45,1.60 | 0.99 | 0.94,1.05 | 1.14 | 0.98,1.33 |
| Secondary education | 1.42 | 1.37,1.46 | 1.18 | 1.12,1.24 | 1.11 | 1.06,1.17 | 1.05 | 0.90,1.22 |
| Post-secondary education | 1.00 | 1.00,1.00 | 1.00 | 1.00,1.00 | 1.00 | 1.00,1.00 | 1.00 | 1.00,1.00 |
| Household income (2018-2020) |  |  |  |  |  |  |  |  |
| Q1 | 0.37 | 0.35,0.38 | 0.80 | 0.76,0.85 | 0.33 | 0.30,0.35 | 0.74 | 0.61,0.90 |
| Q2 | 0.77 | 0.74,0.80 | 0.88 | 0.83,0.93 | 0.62 | 0.58,0.67 | 0.90 | 0.74,1.09 |
| Q3 | 1.00 | 0.96,1.04 | 0.89 | 0.84,0.95 | 0.88 | 0.83,0.94 | 1.05 | 0.87,1.27 |
| Q4 | 1.05 | 1.01,1.09 | 0.88 | 0.83,0.94 | 0.98 | 0.92,1.04 | 1.01 | 0.84,1.22 |
| Q5 | 1.00 | 1.00,1.00 | 1.00 | 1.00,1.00 | 1.00 | 1.00,1.00 | 1.00 | 1.00,1.00 |
|  |  |  |  |  |  |  |  |  |

*Digital visits include video and chat consultations from 2020 and onwards.

Supplemental Table 17. Sex-stratified Odds Ratios of *in-person visits* and *digital visits* among youth aged 12-22 in Stockholm County 2018-2022. Age-adjusted estimates, CI95%

|  |  |  |  | |  | |  | | |  |  | | |
| --- | --- | --- | --- | --- | --- | --- | --- | --- | --- | --- | --- | --- | --- |
|  | **In-person visits** | | | | | | | **Digital visits*** | | | | | |
|  | **Females** | | | **Males** | | | | **Females** | | | | **Males** | |
|  | **Adjusted OR** | **CI 95%** | | **Adjusted OR** | | **CI 95%** | | **Adjusted OR** | **CI 95%** | | | **Adjusted OR** | **CI 95%** |
| Age category |  |  | |  | |  | |  |  | | |  |  |
| 12-14 | 0.04 | 0.04,0.04 | | 0.05 | | 0.05,0.06 | | 0.04 | 0.04,0.04 | | | 0.14 | 0.10,0.18 |
| 15-19 | 1.00 | 1.00,1.00 | | 1.00 | | 1.00,1.00 | | 1.00 | 1.00,1.00 | | | 1.00 | 1.00,1.00 |
| 20-22 | 0.97 | 0.96,0.99 | | 1.80 | | 1.74,1.85 | | 1.22 | 1.17,1.27 | | | 1.80 | 1.60,2.03 |
| Migrant background |  |  | |  | |  | |  |  | | |  |  |
| Swedish-born parents | 1.00 | 1.00,1.00 | | 1.00 | | 1.00,1.00 | | 1.00 | 1.00,1.00 | | | 1.00 | 1.00,1.00 |
| Foreign-born parents | 0.29 | 0.28,0.30 | | 1.11 | | 1.05,1.16 | | 0.35 | 0.33,0.38 | | | 1.03 | 0.87,1.22 |
| Foreign-born | 0.17 | 0.16,0.18 | | 0.64 | | 0.61,0.67 | | 0.27 | 0.26,0.29 | | | 0.68 | 0.58,0.80 |
| Birth region |  |  | |  | |  | |  |  | | |  |  |
| Sweden | 1.00 | 1.00,1.00 | | 1.00 | | 1.00,1.00 | | 1.00 | 1.00,1.00 | | | 1.00 | 1.00,1.00 |
| Rest of Europe | 0.24 | 0.23,0.26 | | 0.50 | | 0.46,0.54 | | 0.34 | 0.31,0.38 | | | 0.68 | 0.52,0.90 |
| Rest of world | 0.19 | 0.18,0.20 | | 0.68 | | 0.65,0.72 | | 0.30 | 0.28,0.33 | | | 0.67 | 0.56,0.81 |
| Maternal education level (2018-2020) |  |  | |  | |  | |  |  | | |  |  |
| Primary education | 0.87 | 0.85,0.90 | | 1.17 | | 1.11,1.22 | | 0.60 | 0.57,0.64 | | | 0.85 | 0.73,0.99 |
| Secondary education | 1.25 | 1.21,1.28 | | 1.08 | | 1.03,1.13 | | 0.96 | 0.91,1.00 | | | 0.83 | 0.72,0.96 |
| Post-secondary education | 1.00 | 1.00,1.00 | | 1.00 | | 1.00,1.00 | | 1.00 | 1.00,1.00 | | | 1.00 | 1.00,1.00 |
| Paternal education level (2018-2020) |  |  | |  | |  | |  |  | | |  |  |
| Primary education | 1.30 | 1.26,1.34 | | 1.18 | | 1.13,1.24 | | 0.81 | 0.77,0.85 | | | 0.93 | 0.80,1.08 |
| Secondary education | 1.38 | 1.34,1.43 | | 1.10 | | 1.05,1.15 | | 1.05 | 0.99,1.11 | | | 0.98 | 0.84,1.14 |
| Post-secondary education | 1.00 | 1.00,1.00 | | 1.00 | | 1.00,1.00 | | 1.00 | 1.00,1.00 | | | 1.00 | 1.00,1.00 |
| Household income (2018-2020) |  |  | |  | |  | |  |  | | |  |  |
| Q1 | 0.33 | 0.32,0.35 | | 0.80 | | 0.76,0.85 | | 0.30 | 0.28,0.33 | | | 0.71 | 0.58,0.87 |
| Q2 | 0.77 | 0.74,0.80 | | 0.89 | | 0.84,0.95 | | 0.61 | 0.57,0.65 | | | 0.89 | 0.73,1.08 |
| Q3 | 1.02 | 0.98,1.07 | | 0.90 | | 0.85,0.96 | | 0.87 | 0.82,0.93 | | | 1.05 | 0.87,1.26 |
| Q4 | 1.07 | 1.02,1.11 | | 0.90 | | 0.84,0.95 | | 0.97 | 0.91,1.03 | | | 1.01 | 0.83,1.22 |
| Q5 | 1.00 | 1.00,1.00 | | 1.00 | | 1.00,1.00 | | 1.00 | 1.00,1.00 | | | 1.00 | 1.00,1.00 |

*Digital visits include video and chat consultations data 2020 and onwards.

Supplemental Table 18. Odds Ratios and 95% confidence interval of SRH-use at population level for *video consultations*, 2020-2022 (CI 95%). Crude and age and sex-adjusted estimates.

| Video consultations | Crude OR | CI 95% | Adjusted OR | CI 95% |
| --- | --- | --- | --- | --- |
| Sex |  |  |  |  |
| Females | 1.00 | 1.00,1.00 | 1.00 | 1.00,1.00 |
| Males | 0.01 | 0.01,0.02 | 0.01 | 0.01,0.01 |
| Age category |  |  |  |  |
| 12-14 | 0.04 | 0.03,0.04 | 0.03 | 0.03,0.04 |
| 15-19 | 1.00 | 1.00,1.00 | 1.00 | 1.00,1.00 |
| 20-22 | 1.18 | 1.13,1.23 | 1.22 | 1.17,1.27 |
| Migrant background |  |  |  |  |
| Swedish-born parents | 1.00 | 1.00,1.00 | 1.00 | 1.00,1.00 |
| Foreign-born parents | 0.31 | 0.28,0.33 | 0.30 | 0.28,0.33 |
| Foreign-born | 0.30 | 0.28,0.32 | 0.25 | 0.23,0.27 |
| Birth region |  |  |  |  |
| Sweden | 1.00 | 1.00,1.00 | 1.00 | 1.00,1.00 |
| Rest of Europe | 0.40 | 0.36,0.45 | 0.33 | 0.29,0.37 |
| Rest of world | 0.33 | 0.30,0.36 | 0.27 | 0.25,0.30 |
| Maternal education level (2018-2020) |  |  |  |  |
| Primary education | 0.73 | 0.69,0.78 | 0.56 | 0.53,0.60 |
| Secondary education | 1.08 | 1.03,1.14 | 0.94 | 0.89,0.99 |
| Post-secondary education | 1.00 | 1.00,1.00 | 1.00 | 1.00,1.00 |
| Paternal education level (2018-2020) |  |  |  |  |
| Primary education | 0.96 | 0.90,1.01 | 0.78 | 0.73,0.83 |
| Secondary education | 1.08 | 1.02,1.14 | 1.02 | 0.96,1.08 |
| Post-secondary education | 1.00 | 1.00,1.00 | 1.00 | 1.00,1.00 |
| Household income (2018-2020) |  |  |  |  |
| Q1 | 0.27 | 0.25,0.30 | 0.25 | 0.23,0.27 |
| Q2 | 0.55 | 0.51,0.59 | 0.54 | 0.50,0.59 |
| Q3 | 0.82 | 0.77,0.88 | 0.82 | 0.77,0.88 |
| Q4 | 0.96 | 0.90,1.03 | 0.96 | 0.89,1.02 |
| Q5 | 1.00 | 1.00,1.00 | 1.00 | 1.00,1.00 |
|  |  |  |  |  |

.

Supplemental Table 19. Odds Ratios and 95% confidence interval of SRH-use at population level for *chat consultations*, 2020-2022 (CI 95%). Crude and age and sex-adjusted estimates.

| Chat consultations | Crude OR | CI 95% | Adjusted OR | CI 95% |
| --- | --- | --- | --- | --- |
| Sex |  |  |  |  |
| Females | 1.00 | 1.00,1.00 | 1.00 | 1.00,1.00 |
| Males | 0.14 | 0.13,0.15 | 0.13 | 0.12,0.14 |
| Age category |  |  |  |  |
| 12-14 | 0.07 | 0.06,0.08 | 0.07 | 0.06,0.08 |
| 15-19 | 1.00 | 1.00,1.00 | 1.00 | 1.00,1.00 |
| 20-22 | 1.18 | 1.12,1.25 | 1.21 | 1.15,1.28 |
| Migrant background |  |  |  |  |
| Swedish-born parents | 1.00 | 1.00,1.00 | 1.00 | 1.00,1.00 |
| Foreign-born parents | 0.65 | 0.60,0.71 | 0.68 | 0.62,0.74 |
| Foreign-born | 0.55 | 0.51,0.59 | 0.49 | 0.45,0.53 |
| Birth region |  |  |  |  |
| Sweden | 1.00 | 1.00,1.00 | 1.00 | 1.00,1.00 |
| Rest of Europe | 0.54 | 0.47,0.62 | 0.48 | 0.42,0.55 |
| Rest of world | 0.61 | 0.56,0.67 | 0.54 | 0.50,0.60 |
| Maternal education level (2018-2020) |  |  |  |  |
| Primary education | 1.00 | 0.93,1.07 | 0.82 | 0.76,0.88 |
| Secondary education | 1.11 | 1.04,1.18 | 0.99 | 0.93,1.06 |
| Post-secondary education | 1.00 | 1.00,1.00 | 1.00 | 1.00,1.00 |
| Paternal education level (2018-2020) |  |  |  |  |
| Primary education | 1.09 | 1.02,1.17 | 0.92 | 0.86,0.99 |
| Secondary education | 1.14 | 1.07,1.22 | 1.09 | 1.01,1.17 |
| Post-secondary education | 1.00 | 1.00,1.00 | 1.00 | 1.00,1.00 |
| Household income (2018-2020) |  |  |  |  |
| Q1 | 0.61 | 0.56,0.67 | 0.59 | 0.54,0.65 |
| Q2 | 0.88 | 0.81,0.96 | 0.88 | 0.81,0.96 |
| Q3 | 1.01 | 0.93,1.10 | 1.02 | 0.93,1.11 |
| Q4 | 0.99 | 0.91,1.08 | 0.99 | 0.91,1.08 |
| Q5 | 1.00 | 1.00,1.00 | 1.00 | 1.00,1.00 |
|  |  |  |  |  |

.

Supplemental Table 20. Sex-stratified Odds Ratios of *video consultations* and *chat consultations* among youth aged 12-22 in Stockholm County 2020-2022. Crude estimates, CI95%.

|  | Video consultations | | | | Chat consultations | | | |
| --- | --- | --- | --- | --- | --- | --- | --- | --- |
|  | **Females** | | **Males** | | **Females** | | **Males** | |
|  | **Crude**  **OR** | **CI 95%** | **Crude**  **OR** | **CI 95%** | **Crude OR** | **CI 95%** | **Crude**  **OR** | **CI 95%** |
| Age category |  |  |  |  |  |  |  |  |
| 12-14 | 0.03 | 0.03,0.04 | 0.14 | 0.08,0.25 | 0.06 | 0.05,0.08 | 0.14 | 0.10,0.19 |
| 15-19 | 1.00 | 1.00,1.00 | 1.00 | 1.00,1.00 | 1.00 | 1.00,1.00 | 1.00 | 1.00,1.00 |
| 20-22 | 1.18 | 1.13,1.24 | 2.53 | 2.03,3.14 | 1.15 | 1.08,1.22 | 1.59 | 1.38,1.82 |
| Migrant background |  |  |  |  |  |  |  |  |
| Swedish-born parents | 1.00 | 1.00,1.00 | 1.00 | 1.00,1.00 | 1.00 | 1.00,1.00 | 1.00 | 1.00,1.00 |
| Foreign-born parents | 0.29 | 0.27,0.31 | 0.80 | 0.43,1.50 | 0.59 | 0.54,0.64 | 1.07 | 0.88,1.29 |
| Foreign-born | 0.29 | 0.27,0.32 | 0.80 | 0.45,1.40 | 0.52 | 0.47,0.56 | 0.88 | 0.73,1.06 |
| Birth region |  |  |  |  |  |  |  |  |
| Sweden | 1.00 | 1.00,1.00 | 1.00 | 1.00,1.00 | 1.00 | 1.00,1.00 | 1.00 | 1.00,1.00 |
| Rest of Europe | 0.38 | 0.34,0.43 | 0.63 | 0.21,1.84 | 0.49 | 0.42,0.57 | 0.82 | 0.59,1.13 |
| Rest of world | 0.33 | 0.30,0.36 | 0.91 | 0.49,1.70 | 0.60 | 0.54,0.66 | 0.89 | 0.72,1.09 |
| Maternal education level (2018-2020) |  |  |  |  |  |  |  |  |
| Primary education | 0.71 | 0.67,0.76 | 1.27 | 0.74,2.16 | 0.99 | 0.92,1.07 | 1.00 | 0.83,1.20 |
| Secondary education | 1.08 | 1.02,1.14 | 1.04 | 0.63,1.72 | 1.14 | 1.07,1.22 | 0.91 | 0.77,1.08 |
| Post-secondary education | 1.00 | 1.00,1.00 | 1.00 | 1.00,1.00 | 1.00 | 1.00,1.00 | 1.00 | 1.00,1.00 |
| Paternal education level (2018-2020) |  |  |  |  |  |  |  |  |
| Primary education | 0.94 | 0.89,1.00 | 1.27 | 0.74,2.16 | 1.09 | 1.01,1.18 | 1.06 | 0.89,1.27 |
| Secondary education | 1.08 | 1.02,1.15 | 1.04 | 0.63,1.72 | 1.16 | 1.08,1.25 | 1.04 | 0.87,1.24 |
| Post-secondary education | 1.00 | 1.00,1.00 | 1.00 | 1.00,1.00 | 1.00 | 1.00,1.00 | 1.00 | 1.00,1.00 |
| Household income (2018-2020) |  |  |  |  |  |  |  |  |
| Q1 | 0.26 | 0.24,0.28 | 0.70 | 0.49,1.00 | 0.59 | 0.53,0.65 | 0.78 | 0.61,0.98 |
| Q2 | 0.55 | 0.51,0.59 | 0.74 | 0.52,1.06 | 0.87 | 0.79,0.96 | 0.98 | 0.78,1.22 |
| Q3 | 0.82 | 0.77,0.88 | 1.04 | 0.74,1.44 | 1.01 | 0.92,1.11 | 1.08 | 0.86,1.35 |
| Q4 | 0.96 | 0.90,1.03 | 1.08 | 0.78,1.50 | 0.98 | 0.90,1.08 | 1.02 | 0.82,1.28 |
| Q5 | 1.00 | 1.00,1.00 | 1.00 | 1.00,1.00 | 1.00 | 1.00,1.00 | 1.00 | 1.00,1.00 |
|  |  |  |  |  |  |  |  |  |

.

Supplemental Table 21. Sex-stratified Odds Ratios of *video consultations* and *chat consultations* among youth aged 12-22 in Stockholm County 2020-2022. Age-adjusted estimates, CI95%.

|  | Video consultations | | | | Chat consultations | | | |
| --- | --- | --- | --- | --- | --- | --- | --- | --- |
|  | **Males** | | **Females** | | **Males** | | **Females** | |
|  | **Adjusted OR** | **CI 95%** | **Adjusted OR** | **CI 95%** | **Adjusted OR** | **CI 95%** | **Adjusted OR** | **CI 95%** |
| Age category |  |  |  |  |  |  |  |  |
| 12-14 | 0.03 | 0.03,0.04 | 0.14 | 0.08,0.25 | 0.06 | 0.05,0.08 | 0.14 | 0.10,0.19 |
| 15-19 | 1.00 | 1.00,1.00 | 1.00 | 1.00,1.00 | 1.00 | 1.00,1.00 | 1.00 | 1.00,1.00 |
| 20-22 | 1.18 | 1.13,1.24 | 2.53 | 2.03,3.14 | 1.15 | 1.08,1.22 | 1.59 | 1.38,1.82 |
| Migrant background |  |  |  |  |  |  |  |  |
| Swedish-born parents | 1.00 | 1.00,1.00 | 1.00 | 1.00,1.00 | 1.00 | 1.00,1.00 | 1.00 | 1.00,1.00 |
| Foreign-born parents | 0.29 | 0.26,0.31 | 0.82 | 0.59,1.12 | 0.61 | 0.55,0.67 | 1.11 | 0.92,1.34 |
| Foreign-born | 0.24 | 0.22,0.26 | 0.59 | 0.44,0.79 | 0.45 | 0.41,0.49 | 0.72 | 0.60,0.87 |
| Birth region |  |  |  |  |  |  |  |  |
| Sweden | 1.00 | 1.00,1.00 | 1.00 | 1.00,1.00 | 1.00 | 1.00,1.00 | 1.00 | 1.00,1.00 |
| Rest of Europe | 0.32 | 0.28,0.36 | 0.57 | 0.34,0.98 | 0.44 | 0.37,0.51 | 0.72 | 0.53,0.99 |
| Rest of world | 0.26 | 0.24,0.29 | 0.62 | 0.45,0.86 | 0.51 | 0.46,0.56 | 0.70 | 0.57,0.86 |
| Maternal education level (2018-2020) |  |  |  |  |  |  |  |  |
| Primary education | 0.55 | 0.51,0.59 | 0.97 | 0.74,1.27 | 0.82 | 0.76,0.89 | 0.81 | 0.68,0.97 |
| Secondary education | 0.94 | 0.89,0.99 | 0.91 | 0.70,1.18 | 1.03 | 0.96,1.10 | 0.81 | 0.69,0.96 |
| Post-secondary education | 1.00 | 1.00,1.00 | 1.00 | 1.00,1.00 | 1.00 | 1.00,1.00 | 1.00 | 1.00,1.00 |
| Paternal education level (2018-2020) |  |  |  |  |  |  |  |  |
| Primary education | 0.77 | 0.72,0.82 | 0.97 | 0.74,1.27 | 0.93 | 0.86,1.01 | 0.88 | 0.73,1.05 |
| Secondary education | 1.02 | 0.96,1.08 | 0.91 | 0.70,1.18 | 1.11 | 1.03,1.20 | 0.97 | 0.81,1.15 |
| Post-secondary education | 1.00 | 1.00,1.00 | 1.00 | 1.00,1.00 | 1.00 | 1.00,1.00 | 1.00 | 1.00,1.00 |
| Household income (2018-2020) |  |  |  |  |  |  |  |  |
| Q1 | 0.24 | 0.22,0.26 | 0.67 | 0.46,0.95 | 0.56 | 0.51,0.62 | 0.75 | 0.59,0.94 |
| Q2 | 0.54 | 0.50,0.58 | 0.73 | 0.51,1.04 | 0.87 | 0.79,0.95 | 0.96 | 0.77,1.20 |
| Q3 | 0.81 | 0.76,0.87 | 1.02 | 0.74,1.43 | 1.00 | 0.92,1.10 | 1.08 | 0.87,1.34 |
| Q4 | 0.95 | 0.89,1.02 | 1.08 | 0.78,1.50 | 0.98 | 0.89,1.08 | 1.02 | 0.82,1.28 |
| Q5 | 1.00 | 1.00,1.00 | 1.00 | 1.00,1.00 | 1.00 | 1.00,1.00 | 1.00 | 1.00,1.00 |

.

**
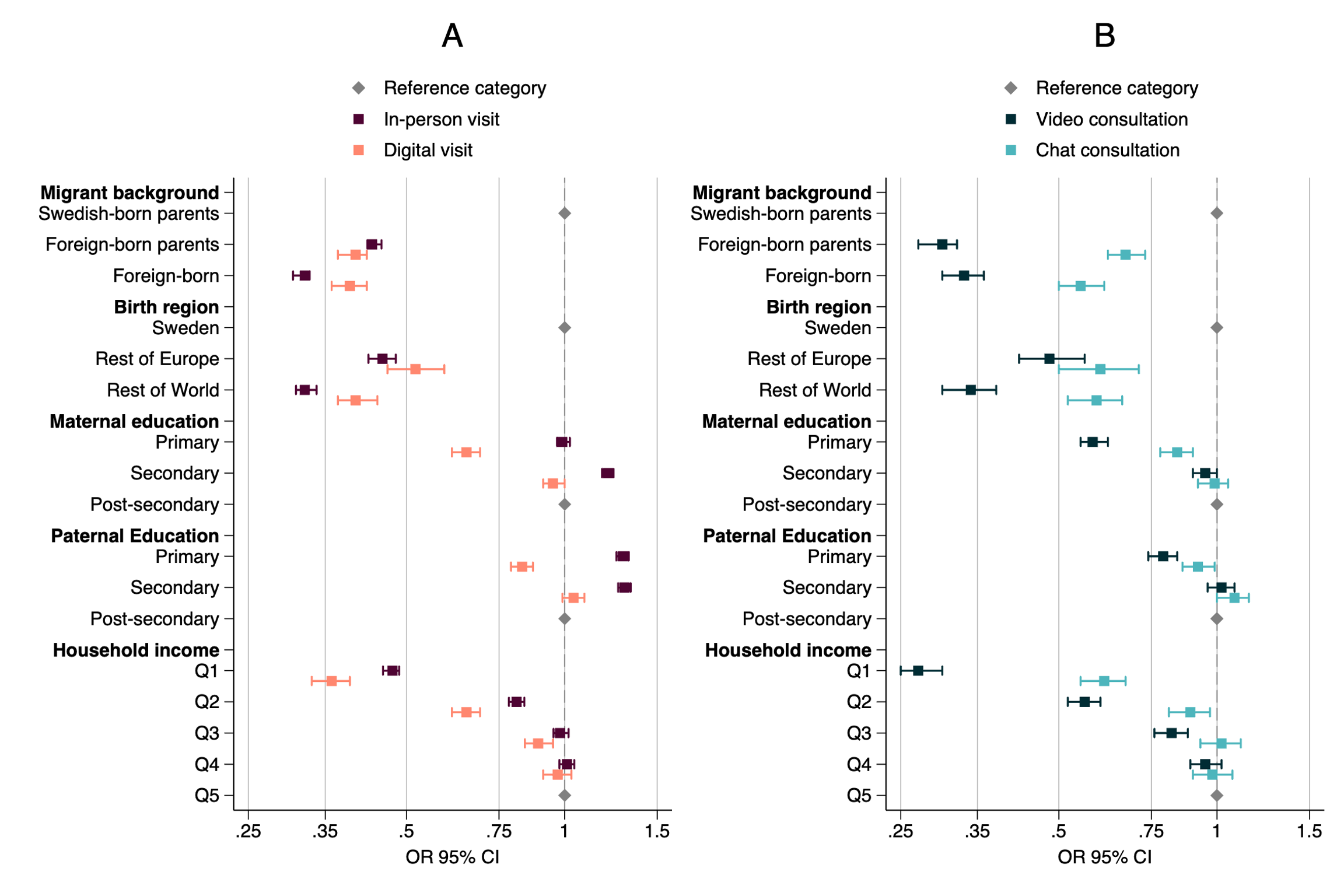
**

Supplemental Figure 7. Odds Ratios and 95% confidence interval of SRH-contacts at population level for in-person and digital visits across the entire study period. Sensitivity analysis of *complete cases.* Estimates are age adjusted. Error bars represent 95% confidence intervals.

Supplemental Table 22. Annual utilization rate (%) of *in-person visits* among youth aged 12-22 in Stockholm County 2018-2022. Sensitivity analysis of *complete cases.* Crude and age and sex-adjusted estimates, CI95%.

| In-person visits | Crude estimates (%) | CI 95% | Adjusted estimates (%) | CI 95% |
| --- | --- | --- | --- | --- |
| Sex |  |  |  |  |
| Females | 25.02 | 24.88,25.17 | 16.47 | 16.32,16.62 |
| Males | 3.83 | 3.77,3.89 | 1.78 | 1.75,1.82 |
| Age category |  |  |  |  |
| 12-14 | 2.78 | 2.72,2.85 | 1.17 | 1.14,1.20 |
| 15-19 | 18.55 | 18.43,18.67 | 12.03 | 11.92,12.14 |
| 20-22 | 19.64 | 19.49,19.78 | 13.62 | 13.48,13.75 |
| Migrant background |  |  |  |  |
| Swedish-born parents | 16.72 | 16.61,16.84 | 6.10 | 6.03,6.18 |
| Foreign-born parents | 10.61 | 10.42,10.81 | 3.43 | 3.35,3.51 |
| Foreign-born | 9.32 | 9.10,9.53 | 2.71 | 2.64,2.79 |
| Birth region |  |  |  |  |
| Sweden | 15.68 | 15.58,15.78 | 5.61 | 5.54,5.67 |
| Rest of Europe | 9.99 | 9.62,10.38 | 3.21 | 3.07,3.36 |
| Rest of world | 9.06 | 8.81,9.32 | 2.51 | 2.43,2.60 |
| Maternal education level (2018-2020) |  |  |  |  |
| Primary education | 15.62 | 15.44,15.80 | 5.02 | 4.93,5.11 |
| Secondary education | 16.09 | 15.94,16.25 | 5.71 | 5.63,5.80 |
| Post-secondary education | 13.62 | 13.48,13.76 | 5.04 | 4.96,5.11 |
| Paternal education level (2018-2020) |  |  |  |  |
| Primary education | 16.44 | 16.29,16.60 | 5.54 | 5.45,5.62 |
| Secondary education | 15.25 | 15.10,15.41 | 5.56 | 5.48,5.65 |
| Post-secondary education | 13.08 | 12.93,13.24 | 4.67 | 4.59,4.75 |
| Household income (2018-2020) |  |  |  |  |
| Q1 | 10.90 | 10.71,11.09 | 3.48 | 3.40,3.55 |
| Q2 | 14.44 | 14.24,14.64 | 5.10 | 5.01,5.20 |
| Q3 | 15.95 | 15.75,16.15 | 5.81 | 5.70,5.92 |
| Q4 | 16.42 | 16.22,16.62 | 5.91 | 5.80,6.01 |
| Q5 | 16.47 | 16.27,16.67 | 5.87 | 5.77,5.98 |

Supplemental Table 23. Annual utilization rate (%) of *digital visits* among youth aged 12-22 in Stockholm County 2020-2022. Sensitivity analysis of *complete cases*. Crude and age and sex-adjusted estimates, CI95%.

| Digital visits* | Crude estimates (%) | CI 95% | Adjusted estimates (%) | CI 95% |
| --- | --- | --- | --- | --- |
| Sex |  |  |  |  |
| Females | 4.71 | 4.63,4.79 | 2.54 | 2.47,2.62 |
| Males | 0.29 | 0.27,0.30 | 0.13 | 0.12,0.14 |
| Age category |  |  |  |  |
| 12-14 | 0.21 | 0.19,0.23 | 0.08 | 0.07,0.09 |
| 15-19 | 2.98 | 2.92,3.04 | 1.37 | 1.32,1.42 |
| 20-22 | 3.77 | 3.68,3.86 | 1.77 | 1.71,1.84 |
| Migrant background |  |  |  |  |
| Swedish-born parents | 2.84 | 2.79,2.89 | 0.66 | 0.64,0.69 |
| Foreign-born parents | 1.31 | 1.25,1.38 | 0.29 | 0.27,0.31 |
| Foreign-born | 1.39 | 1.31,1.48 | 0.28 | 0.26,0.31 |
| Birth region |  |  |  |  |
| Sweden | 2.55 | 2.51,2.60 | 0.59 | 0.57,0.62 |
| Rest of Europe | 1.49 | 1.35,1.64 | 0.33 | 0.29,0.37 |
| Rest of world | 1.35 | 1.25,1.45 | 0.26 | 0.24,0.29 |
| Maternal education level (2018-2020) |  |  |  |  |
| Primary education | 2.08 | 2.01,2.16 | 0.42 | 0.39,0.44 |
| Secondary education | 2.60 | 2.53,2.67 | 0.58 | 0.56,0.61 |
| Post-secondary education | 2.44 | 2.38,2.50 | 0.61 | 0.58,0.64 |
| Paternal education level (2018-2020) |  |  |  |  |
| Primary education | 2.35 | 2.29,2.42 | 0.49 | 0.47,0.52 |
| Secondary education | 2.53 | 2.46,2.60 | 0.60 | 0.57,0.63 |
| Post-secondary education | 2.35 | 2.28,2.42 | 0.58 | 0.55,0.61 |
| Household income (2018-2020) |  |  |  |  |
| Q1 | 1.33 | 1.27,1.40 | 0.28 | 0.26,0.30 |
| Q2 | 2.08 | 2.01,2.17 | 0.48 | 0.45,0.50 |
| Q3 | 2.68 | 2.59,2.77 | 0.63 | 0.60,0.66 |
| Q4 | 2.90 | 2.80,2.99 | 0.68 | 0.64,0.71 |
| Q5 | 2.96 | 2.86,3.05 | 0.70 | 0.66,0.73 |

*Digital visits include video and chat consultations from 2020 and onwards.

Supplemental Table 24. Odds Ratios and 95% confidence interval of SRH-use at population level for *in-person visits*, 2018-2022 (CI 95%). Sensitivity analysis of *complete cases*. Crude and age and sex-adjusted estimates.

| In-person visits | Crude estimates (%) | CI 95% | Adjusted estimates (%) | CI 95% |
| --- | --- | --- | --- | --- |
| Sex |  |  |  |  |
| Females | 1.00 | 1.00, 1.00 | 1.00 | 1.00, 1.00 |
| Males | 0.05 | 0.05, 0.05 | 0.04 | 0.04, 0.04 |
| Age category |  |  |  |  |
| 12-14 | 0.04 | 0.04, 0.04 | 0.04 | 0.04, 0.05 |
| 15-19 | 1.00 | 1.00, 1.00 | 1.00 | 1.00, 1.00 |
| 20-22 | 1.13 | 1.11, 1.15 | 1.23 | 1.21, 1.25 |
| Migrant background |  |  |  |  |
| Swedish-born parents | 1.00 | 1.00, 1.00 | 1.00 | 1.00, 1.00 |
| Foreign-born parents | 0.42 | 0.40, 0.43 | 0.43 | 0.42, 0.45 |
| Foreign-born | 0.33 | 0.32, 0.34 | 0.32 | 0.30, 0.33 |
| Birth region |  |  |  |  |
| Sweden | 1.00 | 1.00, 1.00 | 1.00 | 1.00, 1.00 |
| Rest of Europe | 0.43 | 0.40, 0.46 | 0.45 | 0.42, 0.48 |
| Rest of world | 0.36 | 0.34, 0.38 | 0.32 | 0.31, 0.34 |
| Maternal education level (2018-2020) |  |  |  |  |
| Primary education | 1.32 | 1.28, 1.36 | 0.99 | 0.97, 1.02 |
| Secondary education | 1.40 | 1.36, 1.44 | 1.21 | 1.18, 1.24 |
| Post-secondary education | 1.00 | 1.00, 1.00 | 1.00 | 1.00, 1.00 |
| Paternal education level (2018-2020) |  |  |  |  |
| Primary education | 1.59 | 1.54, 1.63 | 1.29 | 1.25, 1.32 |
| Secondary education | 1.35 | 1.32, 1.40 | 1.30 | 1.26, 1.34 |
| Post-secondary education | 1.00 | 1.00, 1.00 | 1.00 | 1.00, 1.00 |
| Household income (2018-2020) |  |  |  |  |
| Q1 | 0.45 | 0.43, 0.47 | 0.47 | 0.45, 0.48 |
| Q2 | 0.76 | 0.74, 0.79 | 0.81 | 0.78, 0.84 |
| Q3 | 0.93 | 0.90, 0.97 | 0.98 | 0.95, 1.02 |
| Q4 | 0.99 | 0.96, 1.03 | 1.01 | 0.98, 1.04 |
| Q5 | 1.00 | 1.00, 1.00 | 1.00 | 1.00, 1.00 |

.´

Supplemental Table 25. Odds Ratios and 95% confidence interval of SRH-use at population level for *digital visits*, 2020-2022 (CI 95%). Sensitivity analysis of *complete cases*. Crude and age and sex-adjusted estimates.

| Digital visits* | Crude estimates (%) | CI 95% | Adjusted estimates (%) | CI 95% |
| --- | --- | --- | --- | --- |
| Sex |  |  |  |  |
| Females | 1.00 | 1.00,1.00 | 1.00 | 1.00,1.00 |
| Males | 0.04 | 0.04,0.04 | 0.04 | 0.03,0.04 |
| Age category |  |  |  |  |
| 12-14 | 0.05 | 0.04,0.05 | 0.05 | 0.04,0.05 |
| 15-19 | 1.00 | 1.00,1.00 | 1.00 | 1.00,1.00 |
| 20-22 | 1.36 | 1.31,1.42 | 1.35 | 1.30,1.41 |
| Migrant background |  |  |  |  |
| Swedish-born parents | 1.00 | 1.00,1.00 | 1.00 | 1.00,1.00 |
| Foreign-born parents | 0.39 | 0.36,0.41 | 0.40 | 0.37,0.42 |
| Foreign-born | 0.41 | 0.38,0.45 | 0.39 | 0.36,0.42 |
| Birth region |  |  |  |  |
| Sweden | 1.00 | 1.00,1.00 | 1.00 | 1.00,1.00 |
| Rest of Europe | 0.51 | 0.45,0.58 | 0.52 | 0.46,0.59 |
| Rest of world | 0.46 | 0.42,0.50 | 0.40 | 0.37,0.44 |
| Maternal education level (2018-2020) |  |  |  |  |
| Primary education | 0.82 | 0.78,0.86 | 0.65 | 0.61,0.69 |
| Secondary education | 1.09 | 1.04,1.14 | 0.95 | 0.91,1.00 |
| Post-secondary education | 1.00 | 1.00,1.00 | 1.00 | 1.00,1.00 |
| Paternal education level (2018-2020) |  |  |  |  |
| Primary education | 1.00 | 0.95,1.05 | 0.83 | 0.79,0.87 |
| Secondary education | 1.10 | 1.04,1.15 | 1.04 | 0.99,1.09 |
| Post-secondary education | 1.00 | 1.00,1.00 | 1.00 | 1.00,1.00 |
| Household income (2018-2020) |  |  |  |  |
| Q1 | 0.37 | 0.35,0.40 | 0.36 | 0.33,0.39 |
| Q2 | 0.64 | 0.60,0.68 | 0.65 | 0.61,0.69 |
| Q3 | 0.88 | 0.83,0.93 | 0.89 | 0.84,0.95 |
| Q4 | 0.97 | 0.92,1.03 | 0.97 | 0.91,1.03 |
| Q5 | 1.00 | 1.00,1.00 | 1.00 | 1.00,1.00 |

*Digital visits include video and chat consultations data from 2020 and onwards.

.

Supplemental Table 26. Odds Ratios and 95% confidence interval of SRH-use at population level for *video consultations*, 2020-2022 (CI 95%). Sensitivity analysis of *complete cases*. Crude and age and sex-adjusted estimates.

| Video consultations 2020-2022* | Crude OR | CI 95% | Adjusted OR | CI 95% |
| --- | --- | --- | --- | --- |
| Sex |  |  |  |  |
| Females | 1.00 | 1.00,1.00 | 1.00 | 1.00,1.00 |
| Males | 0.01 | 0.01,0.02 | 0.01 | 0.01,0.01 |
| Age category |  |  |  |  |
| 12-14 | 0.04 | 0.03,0.04 | 0.04 | 0.03,0.04 |
| 15-19 | 1.00 | 1.00,1.00 | 1.00 | 1.00,1.00 |
| 20-22 | 1.31 | 1.25,1.37 | 1.31 | 1.25,1.37 |
| Migrant background |  |  |  |  |
| Swedish-born parents | 1.00 | 1.00,1.00 | 1.00 | 1.00,1.00 |
| Foreign-born parents | 0.30 | 0.28,0.33 | 0.30 | 0.27,0.32 |
| Foreign-born | 0.36 | 0.33,0.40 | 0.33 | 0.30,0.36 |
| Birth region |  |  |  |  |
| Sweden | 1.00 | 1.00,1.00 | 1.00 | 1.00,1.00 |
| Rest of Europe | 0.49 | 0.42,0.56 | 0.48 | 0.42,0.56 |
| Rest of world | 0.40 | 0.36,0.44 | 0.34 | 0.30,0.38 |
| Maternal education level (2018-2020) |  |  |  |  |
| Primary education | 0.74 | 0.70,0.79 | 0.58 | 0.55,0.62 |
| Secondary education | 1.09 | 1.03,1.14 | 0.95 | 0.90,1.00 |
| Post-secondary education | 1.00 | 1.00,1.00 | 1.00 | 1.00,1.00 |
| Paternal education level (2018-2020) |  |  |  |  |
| Primary education | 0.95 | 0.90,1.01 | 0.79 | 0.74,0.84 |
| Secondary education | 1.07 | 1.01,1.14 | 1.02 | 0.96,1.08 |
| Post-secondary education | 1.00 | 1.00,1.00 | 1.00 | 1.00,1.00 |
| Household income (2018-2020) |  |  |  |  |
| Q1 | 0.29 | 0.27,0.32 | 0.27 | 0.25,0.30 |
| Q2 | 0.56 | 0.52,0.60 | 0.56 | 0.52,0.60 |
| Q3 | 0.82 | 0.76,0.87 | 0.82 | 0.76,0.88 |
| Q4 | 0.96 | 0.89,1.02 | 0.95 | 0.89,1.02 |
| Q5 | 1.00 | 1.00,1.00 | 1.00 | 1.00,1.00 |

.

Supplemental Table 27. Odds Ratios and 95% confidence interval of SRH-use at population level for *chat consultations*, 2020-2022 (CI 95%). Sensitivity analysis of *complete cases*. Crude and age and sex-adjusted estimates.

| Chat consultations 2020-2022* | Crude OR | CI 95% | Adjusted OR | CI 95% |
| --- | --- | --- | --- | --- |
| Sex |  |  |  |  |
| Females | 1.00 | 1.00,1.00 | 1.00 | 1.00,1.00 |
| Males | 0.13 | 0.12,0.14 | 0.12 | 0.12,0.13 |
| Age category |  |  |  |  |
| 12-14 | 0.08 | 0.07,0.09 | 0.08 | 0.07,0.09 |
| 15-19 | 1.00 | 1.00,1.00 | 1.00 | 1.00,1.00 |
| 20-22 | 1.28 | 1.21,1.35 | 1.28 | 1.21,1.35 |
| Migrant background |  |  |  |  |
| Swedish-born parents | 1.00 | 1.00,1.00 | 1.00 | 1.00,1.00 |
| Foreign-born parents | 0.66 | 0.60,0.71 | 0.67 | 0.62,0.73 |
| Foreign-born | 0.59 | 0.53,0.65 | 0.55 | 0.50,0.61 |
| Birth region |  |  |  |  |
| Sweden | 1.00 | 1.00,1.00 | 1.00 | 1.00,1.00 |
| Rest of Europe | 0.59 | 0.50,0.70 | 0.60 | 0.50,0.71 |
| Rest of world | 0.65 | 0.57,0.73 | 0.59 | 0.52,0.66 |
| Maternal education level (2018-2020) |  |  |  |  |
| Primary education | 1.00 | 0.93,1.08 | 0.84 | 0.78,0.90 |
| Secondary education | 1.10 | 1.03,1.17 | 0.99 | 0.92,1.05 |
| Post-secondary education | 1.00 | 1.00,1.00 | 1.00 | 1.00,1.00 |
| Paternal education level (2018-2020) |  |  |  |  |
| Primary education | 1.07 | 1.00,1.15 | 0.92 | 0.86,0.99 |
| Secondary education | 1.13 | 1.05,1.21 | 1.08 | 1.00,1.15 |
| Post-secondary education | 1.00 | 1.00,1.00 | 1.00 | 1.00,1.00 |
| Household income (2018-2020) |  |  |  |  |
| Q1 | 0.63 | 0.57,0.69 | 0.61 | 0.55,0.67 |
| Q2 | 0.88 | 0.81,0.96 | 0.89 | 0.81,0.97 |
| Q3 | 1.01 | 0.93,1.10 | 1.02 | 0.93,1.11 |
| Q4 | 0.98 | 0.90,1.07 | 0.98 | 0.90,1.07 |
| Q5 | 1.00 | 1.00,1.00 | 1.00 | 1.00,1.00 |

.


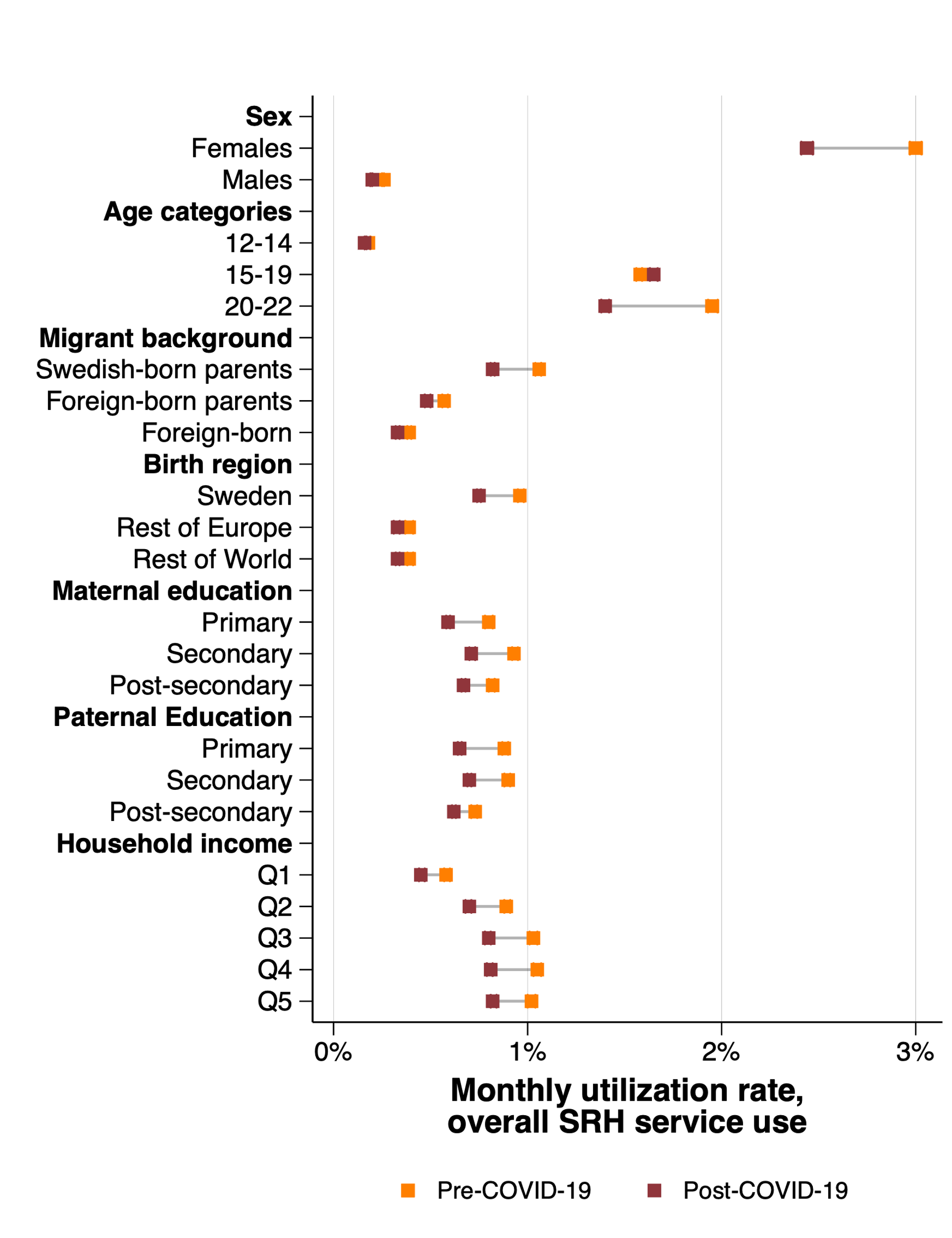


Supplemental Figure 8. Adjusted differences in the percent of monthly overall Sexual and Reproductive Health (SRH) visits at youth clinics *within* sociodemographic strata, before and after the COVID-19 pandemic. Estimates are sex and age-adjusted, except when analyzed as the strata. 95% confidence intervals are plotted but because of the large sample size, they are not visible beyond the point estimate.

Supplemental Table 28. Differences in percent of monthly overall Sexual and Reproductive Health (SRH) service use at youth clinics *across* sociodemographic strata, before and after the COVID-19 pandemic. Estimates demonstrate relative differences.

|  | Crude | | | | Adjusted | | | |
| --- | --- | --- | --- | --- | --- | --- | --- | --- |
|  | **Pre-COVID-19** | | **Post-COVID-19** | | **Pre-COVID-19** | | **Post-COVID-19** | |
|  | **%** | **CI** | **%** | **CI** | **%** | **CI** | **%** | **CI** |
| Sex |  |  |  |  |  |  |  |  |
| Females | 0.00 | 0.00,0.00 | 0.00 | 0.00,0.00 | 0.00 | 0.00,0.00 | 0.00 | 0.00,0.00 |
| Males | -3.54 | -3.51,-3.58 | -4.09 | -4.05,-4.12 | -4.34 | -4.31,-4.38 | -3.63 | -3.60,-3.66 |
| Age category |  |  |  |  |  |  |  |  |
| 12-14 | -2.53 | -2.50,-2.56 | -2.80 | -2.77,-2.83 | -2.44 | -2.41,-2.47 | -2.59 | -2.56,-2.62 |
| 15-19 | 0.00 | 0.00,0.00 | 0.00 | 0.00,0.00 | 0.00 | 0.00,0.00 | 0.00 | 0.00,0.00 |
| 20-22 | -0.57 | -0.51,-0.62 | -0.55 | -0.52,-0.58 | -0.38 | -0.34,-0.43 | -0.42 | -0.39,-0.45 |
| Migrant background |  |  |  |  |  |  |  |  |
| Swedish-born parents | 0.00 | 0.00,0.00 | 0.00 | 0.00,0.00 | 0.00 | 0.00,0.00 | 0.00 | 0.00,0.00 |
| Foreign-born parents | -1.30 | -1.27,-1.35 | -1.43 | -1.39,-1.46 | -1.32 | -1.28,-1.35 | -0.95 | -0.91,-0.99 |
| Foreign-born | -1.63 | -1.59,-1.67 | -1.86 | -1.82,-1.88 | -1.87 | -1.83,-1.91 | -1.43 | -1.40,-1.46 |
| Birth region |  |  |  |  |  |  |  |  |
| Sweden | 0.00 | 0.00,0.00 | 0.00 | 0.00,0.00 | 0.00 | 0.00,0.00 | 0.00 | 0.00,0.00 |
| Rest of Europe | -1.40 | -1.38,-1.42 | -1.56 | -1.53,-1.59 | -1.63 | -1.58,-1.68 | -1.25 | -1.21,-1.28 |
| Rest of world | -1.39 | -1.34,-1.43 | -1.62 | -1.59,-1.65 | -1.62 | -1.58,-1.65 | -1.25 | -1.21,-1.28 |
| Maternal education level (2018-2020) |  |  |  |  |  |  |  |  |
| Primary education | -0.62 | -0.57,-0.67 | -0.80 | -0.75,-0.85 | 0.05 | 0.00,0.10 | 0.23 | 0.19,0.27 |
| Secondary education | -0.37 | -0.32,-0.42 | -0.50 | -0.45,-0.55 | -0.32 | -0.27,-0.37 | -0.14 | -0.12,-0.16 |
| Post-secondary education | 0.00 | 0.00,0.00 | 0.00 | 0.00,0.00 | 0.00 | 0.00,0.00 | 0.00 | 0.00,0.00 |
| Paternal education level (2018-2020) |  |  |  |  |  |  |  |  |
| Primary education | -0.92 | -0.87,-0.97 | -0.56 | -0.51,-0.61 | -0.44 | -0.39,-0.49 | -0.10 | -0.06,-0.14 |
| Secondary education | -0.45 | -0.41,-0.49 | -0.56 | -0.52,-0.59 | -0.51 | -0.46,-0.54 | -0.24 | -0.20,-0.28 |
| Post-secondary education | 0.00 | 0.00,0.00 | 0.00 | 0.00,0.00 | 0.00 | 0.00,0.00 | 0.00 | 0.00,0.00 |
| Household income (2018-2020) |  |  |  |  |  |  |  |  |
| Q1 | -1.26 | -1.20,-1.30 | -1.52 | -1.46,-1.58 | -1.20 | -1.13,-1.27 | -1.06 | -1.01,-1.11 |
| Q2 | -0.43 | -0.36,-0.49 | -0.56 | -0.49,-0.61 | -0.33 | -0.26,-0.38 | -0.36 | -0.30,-0.39 |
| Q3 | -0.07 | 0.00,-0.14 | -0.17 | -0.10,-0.24 | -0.04 | 0.00,-0.07 | -0.07 | -0.03,-0.13 |
| Q4 | -0.03 | 0.00,-0.04 | -0.07 | 0.00,-0.14 | -0.08 | 0.00,-0.15 | -0.05 | 0.00,-0.10 |
| Q5 | 0.00 | 0.00,0.00 | 0.00 | 0.00,0.00 | 0.00 | 0.00,0.00 | 0.00 | 0.00,0.00 |
|  |  |  |  |  |  |  |  |  |

Supplemental Table 29. Differences in percent of monthly overall Sexual and Reproductive Health (SRH) service use at youth clinics *within* sociodemographic strata, before and after the COVID-19 pandemic. Estimates demonstrate absolute differences.

|  | Crude | | | | Adjusted | | | |
| --- | --- | --- | --- | --- | --- | --- | --- | --- |
|  | **Pre-COVID-19** | | **Post-COVID-19** | | **Pre-COVID-19** | | **Post-COVID-19** | |
|  | **%** | **CI** | **%** | **CI** | **%** | **CI** | **%** | **CI** |
| Sex |  |  |  |  |  |  |  |  |
| Females | 4.06 | 4.03,4.09 | 4.55 | 4.52,4.59 | 3.00 | 2.97,3.03 | 2.44 | 2.41,2.47 |
| Males | 0.45 | 0.44,0.46 | 0.38 | 0.37,0.39 | 0.26 | 0.25,0.26 | 0.20 | 0.19,0.20 |
| Age category |  |  |  |  |  |  |  |  |
| 12-14 | 0.44 | 0.42,0.45 | 0.38 | 0.37,0.40 | 0.18 | 0.17,0.19 | 0.16 | 0.16,0.17 |
| 15-19 | 3.00 | 2.97,3.03 | 3.22 | 3.19,3.25 | 1.58 | 1.56,1.59 | 1.65 | 1.63,1.67 |
| 20-22 | 3.57 | 3.53,3.61 | 2.67 | 2.64,2.70 | 1.95 | 1.93,1.98 | 1.40 | 1.38,1.42 |
| Migrant background |  |  |  |  |  |  |  |  |
| Swedish-born parents | 3.00 | 2.97,3.02 | 3.33 | 3.30,3.35 | 1.06 | 1.04,1.07 | 0.82 | 0.81,0.83 |
| Foreign-born parents | 1.69 | 1.65,1.73 | 1.88 | 1.84,1.93 | 0.57 | 0.56,0.59 | 0.48 | 0.47,0.50 |
| Foreign-born | 1.35 | 1.32,1.39 | 1.45 | 1.41,1.48 | 0.39 | 0.38,0.40 | 0.33 | 0.32,0.34 |
| Birth region |  |  |  |  |  |  |  |  |
| Sweden | 2.77 | 2.75,2.79 | 3.08 | 3.05,3.10 | 0.96 | 0.95,0.98 | 0.75 | 0.74,0.76 |
| Rest of Europe | 1.36 | 1.30,1.42 | 1.50 | 1.44,1.56 | 0.39 | 0.37,0.41 | 0.33 | 0.31,0.34 |
| Rest of world | 1.37 | 1.33,1.41 | 1.45 | 1.41,1.49 | 0.39 | 0.38,0.41 | 0.33 | 0.32,0.34 |
| Maternal education level (2018-2020) |  |  |  |  |  |  |  |  |
| Primary education | 2.86 | 2.82,2.90 | 2.87 | 2.83,2.92 | 0.80 | 0.78,0.82 | 0.59 | 0.58,0.60 |
| Secondary education | 2.87 | 2.83,2.90 | 3.17 | 3.14,3.21 | 0.93 | 0.92,0.95 | 0.71 | 0.70,0.73 |
| Post-secondary education | 2.24 | 2.21,2.27 | 2.68 | 2.64,2.71 | 0.82 | 0.80,0.83 | 0.67 | 0.66,0.68 |
| Paternal education level (2018-2020) |  |  |  |  |  |  |  |  |
| Primary education | 3.02 | 2.98,3.05 | 3.12 | 3.09,3.16 | 0.88 | 0.87,0.90 | 0.65 | 0.64,0.66 |
| Secondary education | 2.65 | 2.61,2.68 | 3.00 | 2.97,3.04 | 0.90 | 0.88,0.91 | 0.70 | 0.69,0.71 |
| Post-secondary education | 2.10 | 2.06,2.13 | 2.57 | 2.53,2.60 | 0.73 | 0.71,0.74 | 0.62 | 0.61,0.63 |
| Household income (2018-2020) |  |  |  |  |  |  |  |  |
| Q1 | 1.84 | 1.80,1.88 | 2.03 | 1.99,2.07 | 0.58 | 0.57,0.60 | 0.45 | 0.44,0.47 |
| Q2 | 2.68 | 2.63,2.73 | 3.00 | 2.95,3.05 | 0.89 | 0.88,0.91 | 0.70 | 0.68,0.71 |
| Q3 | 3.03 | 2.99,3.08 | 3.39 | 3.34,3.44 | 1.03 | 1.01,1.05 | 0.80 | 0.78,0.81 |
| Q4 | 3.14 | 3.09,3.19 | 3.50 | 3.45,3.54 | 1.05 | 1.03,1.07 | 0.81 | 0.79,0.82 |
| Q5 | 3.11 | 3.06,3.15 | 3.56 | 3.51,3.61 | 1.02 | 1.00,1.04 | 0.82 | 0.81,0.84 |
|  |  |  |  |  |  |  |  |  |
