# Supplementary material for: Youth Uptake of Digital Sexual and Reproductive Health Services Across Sociodemographic Groups (2018-2022): A Total Population Study from Stockholm, Sweden
Source: Mayo Clin Proc Digit Health. 2025 Jul 8;3(3):100251. doi: 10.1016/j.mcpdig.2025.100251 (PMC12332933; doi:10.1016/j.mcpdig.2025.100251)
Supplement: Supplementary Data [file mmc2.docx]

Author contributions

### **Title: Youth Uptake of Digital Sexual and Reproductive Health Services Across Sociodemographic Groups (2018-2022): A Total Population Study from Sweden**

- Funding acquisition: KK
- Conceptualization: all authors
- Data curation: GB and LH
- Formal analysis, visualizations, and draft of original manuscript: LH
- Supervision: VHA, AN, AME, & KK
- Writing – review & editing: all authors
- Methodology: all authors
- Validation: LH, GB
